# The association between women’s economic participation and physical and/or sexual domestic violence against women: A case study for Turkey

**DOI:** 10.1371/journal.pone.0273440

**Published:** 2022-11-16

**Authors:** Angela Greulich, Aurélien Dasré

**Affiliations:** 1 I.E.P. Sciences Po Paris - CRIS, Institut Universitaire de France, Paris, France; 2 Université Paris Nanterre-CRESPPA, Nanterre, France; VART Consulting PVT LTD, INDIA

## Abstract

We test in how far women’s economic participation can be associated with physical and/or sexual domestic violence against women in Turkey, by mobilizing the Survey “National Research on Domestic Violence against Women in Turkey” (wave 2014). Several studies found that economically active women have a similar, if not a higher risk of experiencing domestic violence than inactive women in Turkey, as well as in other emerging countries. We challenge these findings for Turkey by distinguishing between formal and informal labor market activities as well as between women who do not work because their partner does not allow them to and women who are inactive for other reasons. To increase the control for endogeneity in this cross-sectional setting, we apply an IV-approach based on cluster averages. We find that, while overall employment for women cannot be associated with a lower risk of experiencing domestic violence for women in Turkey, those women who participate in the *formal* labor market and those women who contribute *at least* the same as their partner to household income are less exposed to physical and/or sexual domestic violence than their counterparts. Distinguishing between formal and informal employment is thus important when it comes to investigate the association between women’s economic activity and domestic violence. This is especially the case in a country like Turkey, which currently undergoes important socio-economic changes and where women in formal and informal employment have therefore very different socioeconomic backgrounds.

## Introduction

The association between women’s economic empowerment and their exposure to domestic violence is ambiguous in the theoretical as well as the empirical literature: not only do economic, sociological and feminist theories disagree with each other, but there is a significant body of empirical research that produces contradictory results. The causes and consequences of domestic violence are complex and interrelated–often interconnected with structural poverty and economic interdependency. Domestic violence against women can thus figure as both a consequence and a cause of asymmetries in the distribution of economic resources between partners.

In several countries, even the most developed ones, economic instability can come along with increases in domestic violence against women. Male unemployment can create stress and tension among partners, and men may fear for their economic supremacy. In the US for example, male unemployment and economic hardship at the household level have increased intimate domestic violence against women during the ‘Great Recession’ [1].

The risk of experiencing domestic violence may be reinforced in countries which are experiencing important economic and social changes (i.e. economic growth combined with an increasing access to higher education and to the formal labor market of lower income groups and women). In Turkey, for example, traditional and modern forces have been struggling with each other for quite a while. The recent economic crisis has reinforced conservative gender and family norms putting in question women’s economic participation. For example, in March 2016, Recep Tayyip Erdoğan has said he believes “a woman is above all else a mother” in a speech marking International Women’s Day. He also said that women are not equal to men and has made proposals to limit abortion rights, the morning-after pill and caesarean sections (The Guardian, 8th March 2016: https://www.theguardian.com/world/2016/mar/08/recep-tayyip-erdogan-a-woman-is-above-all-else-a-mother-turkish-president).

In this article, we investigate the association between women’s economic empowerment and sexual and/or physical domestic violence in Turkey. The co-existence of modern and traditionalist forces make Turkey an interesting study case–especially for other countries that might face similar challenges. For example, women in Turkey have increasing access to higher education, but may still suffer from the cultural attitudes and values towards their employment that is directly related to patriarchal prejudgments [2]. Similarly, Turkey’s current socio-economic transformations may cause conflicts between women’s role in working life and daily family life [3].

To test in how far a woman’s economic empowerment is associated with the risk of experiencing domestic violence in Turkey, we mobilize the Survey “National Research on Domestic Violence against Women in Turkey” (wave 2014). We hereby focus on physical and/or sexual violence experienced by ever-married women (i.e. married at least once) aged 15 to 59, perpetrated by their (last) husband during the last 12 months. We capture two different aspects of female economic empowerment -namely women’s participation in the *formal* labor market and women’s contribution to household income- while we control for important side aspects such as female education and partner characteristics.

In this article, we explicitly distinguish between formal and informal employment for women. This is not always the case in other studies on domestic violence in transition countries. Our results suggest that the way female employment is measured can influence the result to an important extent in Turkey: Several studies find no evidence of a protective role of women’s general labor market participation against domestic violence in countries which currently undergo important economic and social transitions such as Turkey, but also India, Iran, Bangladesh or Jordan [4–11]. We, however, find that in Turkey, those women who are engaged in *formal* labor market activities are less exposed to domestic violence than their counterparts.

The Survey “National Research on Domestic Violence against Women in Turkey” follows international quality standards: it is representative on a national and regional level and has been specially designed to meet the particular needs of this sensitive subject. It provides a large set of reliable information not only on women, but also on partners, on the household and on the family environment. The relatively large sample size (15,072 households) allows econometric specifications, which include a combination of important economic characteristics of women and their partners. This helps disentangling different factors that are potentially linked to domestic violence.

To reduce (albeit not exclude) potential reverse causality between women’s economic participation and domestic violence in this cross-sectional setting, we propose two approaches. First, we distinguish between women whose partner or family does not allow them to work and those who are inactive for other reasons. This makes it possible to disentangle between two types of women: (1) those whose inactivity is likely to emerge as a *consequence* of being with a violent partner and (2) those whose employment status and income contribution can be interpreted as a *determinant* of exposure to domestic violence. Second, we apply an Instrumental Variable approach by using cluster averages of female formal employment and women’s contribution to household income.

The article is structured as follows: Section 2 presents the Turkish context and state of the art on the subject, while section 3 provides information about the data source and measures of domestic violence used in this study. Section 4 presents our methodology, section 5 presents our results and section 6 concludes.

## Context and state of the art

Even though women’s education and employment have risen continuously in Turkey during the last two decades, women still lag behind men in terms of economic empowerment: enrollment rates in higher education are still lower for girls and only a minority of women have access to formal wage employment. In 2013, 58.6% of men aged 20–24 had at least a high school diploma, compared to 48.6% of women, according to the Turkish Demographic Health Survey database (year 2013), and 71% of men aged 15+ participate in the labor force in Turkey, while only 29% of women do. Among the professionally active women, one third work as contributing family workers, while this is only the case for 5% of working men, according to the World Bank World Development Indicators database (year 2014).

Progress in terms of gender equality can be observed in Turkey, but is slow, and over the last decade the public policy discourse has been rather regressive. The current public debate on women’s role in the Turkish society illustrates how much conservative and modernist forces are struggling with each other. While conservatives publicly plead for limiting abortion rights and ask women to concentrate more on their role as mothers, there are an increasing number of legal reforms and policies that seek to promote gender equality and female employment in Turkey. The country’s Tenth Development Plan (2014–18), for example, seeks to facilitate women’s employment through parental leave regulations and investments in formal childcare. The ‘First Action Plan on Women’s Employment in Turkey’ was developed in 2016 to support policies geared to creating decent jobs for women (https://www.ilo.org/ankara/news/WCMS_484712/lang--en/index.htm).

However, conservative gender and family norms are still very dominant, in particular in the Eastern parts of the country, and it seems that traditional gender ideology and patriarchal norms are on the rise in Turkey. For example, legislative changes intending to promote gender equality in employment and in workplaces may not be sufficient in improving women’s labor market position, because domestic responsibilities and other socio-cultural conditions affect women’s employment [3]. In addition, since the economic downturn in 2008, the share of those in Turkey who declare that a university education is more important for a boy than for a girl, and that men should have privileged access to jobs when jobs are scarce, is re-increasing (World Value Surveys 1996, 2001, 2007, 2011).

The dominant conservatism in Turkey supports father and husband leadership, often accompanied by limited freedom of movement for women, which undermines women’s social and economic participation. Despite public efforts to combat domestic violence against women and an increasing awareness of the matter—especially among younger cohorts [12], prevalence rates in domestic violence are still high in comparison to European standards. The prevalence rate of physical and/or sexual domestic violence (in the last 12 months) is 11% in Turkey (Survey “National Research on Domestic Violence Against Women in Turkey, 2014), against 4% on average in the 28 EU countries (Gender-Based Violence against Women Survey Data Set, 2012, from the European Union Agency for Fundamental Rights). Turkey also has one of the highest violence prevalence rates among countries of Eastern Europe and Central Asia [13].

Risk factors for women for experiencing intimate partner violence are multiple. The literature generally distinguishes between two main categories—the bargaining power of women and the propensity for violence of partners (see for example [14] for an overview). In the following, we focus our literature overview on the link between women’s economic empowerment and their risk of experiencing domestic violence carried out by their intimate partners.

### Theories on the impact of women’s economic empowerment on intimate partner violence

Bargaining models of domestic violence predict that women’s economic empowerment, achieved by economic options outside home, improves their power and decision-making inside the household. In addition, women’s financial autonomy increases their capacity to threaten divorce, which has the potential to protect them from domestic violence [15–19]. Higher household income may also reduce spousal violence by reduced economic stress for the family [20]. In addition, higher education might enable women to marry later and chose partners themselves, which increases the chances of finding a non-violent partner. Economic dependence on men is likely to make women vulnerable, not only in financial terms, but also with regards to their physical integrity.

However, economic power and independence of women can also be associated with a high risk of experiencing domestic violence in some cases. A higher relative income position of women can increase their risk of experiencing domestic violence, as male partners may want to appropriate their wife’s income (instrumental theories of violence) or may feel their traditional gender role is threatened [21]. Men may be anxious about losing their breadwinner role and compensate this fear by increasing physical control [22–26]. Colleagues [22] point out that the correlation between female employment and domestic violence is linked with the advancement of gender norms and values in the society. In a highly patriarchal society, where female employment is a rather recent and not yet generalized phenomenon, the risk of domestic violence increasing with female economic empowerment is high. However, the more women’s participation in the formal labor market is accepted and generalized, the more the monetary independence of women will take the lead and protect women from domestic violence. This argument is also mobilized by feminist researchers. They point to the risk of a temporary *backlash* with regard to domestic violence in times of female economic empowerment, as men try to regain control over their wives by using physical and/or sexual violence [27, 28]. The backlash may appear in particular in times of rising male unemployment (i.e. when men experience a sudden change from employment to unemployment), leading women to unexpectedly gain importance in their roles of contributors to household income. At the same time, colleagues [29] explain why an increase in male unemployment does not always increase but can also decrease intimate partner violence. For the authors, the risk of unemployment or being unemployed for men may discourage “latent abusive males” from abusive behaviours as they have an economic interest in avoiding divorce and, therefore, losing spousal insurance. On the contrary, when women face unemployment, women may become more dependent of their partners’ income and therewith less incentive to divorce. Thus, “latent abusive males” may be encouraged to reveal their abusive tendencies when women become unemployed.

Overall, theories on the potential impact of women’s economic empowerment on intimate partner violence suggest a complex relation and put into question a clear correlation between female employment and female income on the one side and women’s risk of experiencing domestic violence on the other side. Dependent of the household, institutional and normative context, the correlation might be positive or at least non-linear due to the risk of male backlash, unexpected changes in relative wages and an opposite effect between the sexes of the change in employment status on domestic violence.

### Overall-empirical evidence on the impact of women’s economic empowerment on intimate partner violence

On the empirical side, the picture is also quite complex. Results depend on if and how the interconnection between domestic violence and the socioeconomic environment is taken into account, as well as on the measures and the methodology which is applied in studying the issue. Another complexity of the issue is that intimate partner violence can have different facets: sexual, physical, psychological and economical.

Studies based on American and Canadian data show that prevalence rates in domestic violence decrease with women’s rising involvement in the formal labor market and with increases in women’s relative wages [16, 19, 24, 30]. However, based on recent US data, colleagues [1] find evidence that incidence rates of domestic violence have increased during the 2001–2010 period (the “Great Recession”) in times of rising male unemployment and economic hardship.

In countries which undergo important economic and social transformations, there is weak evidence that women’s economic empowerment clearly lowers domestic violence, giving credit to backlash theories: Colleagues [31] as well as [7] find, for example, that the prevalence rates of domestic violence are higher for richer women in India. Colleague [8] shows that in India, working women are more exposed to domestic violence than non-working women, especially those who earn more than the partner. Colleagues [9] find similar results for Iran, as do colleagues [10] for rural Bangladesh. By contrast, using nationally representative population-based data from Bangladesh, with adjusted measures of domestic violence, colleagues [32] find that on average higher educated women and those with a higher level of economic autonomy are less likely to report having experienced domestic violence. However, they also find that women who are higher educated and who earn more than their husbands are the ones who have the highest prevalence levels. A recent study based on 66 surveys on 44 countries, including Turkey, identifies a negative cross-country correlation between the proportion of women in the formal workforce and national prevalence rates of intimate partner violence. However, the study also shows that women who participate in the formal labor market have higher risks of experiencing intimate partner violence in countries where few women work in the formal sector [33].

An important methodological concern when studying the impact of female economic empowerment on domestic violence is reverse causality. It is possible that domestic violence influences women’s labor market participation and income contribution [34]. Domestic violence may lead women to seek formal employment outside the house as a strategy of escaping from daily domestic violence, for example. It could also be that women engage in labor market activities to increase their economic independence with the aim of exiting the abusive relationship one day. This mechanism can lead to a positive association between domestic violence against women and the likelihood of women’s employment, which has been showed recently by colleague [35] for Columbia, for example. When empirically assessing the impact of female employment on domestic violence without taking into account this kind of endogeneity, the protective role of formal employment would be underestimated. If, on the contrary, domestic violence hinders women from employment outside the house (due to limited freedom of movement or violated physical integrity, for example, or the intention to hide obvious signs of domestic violence from the outside), the protective role of formal employment would be overestimated.

Only a few studies analysing the economic determinants of domestic violence deal with endogeneity issues. Most of them apply IV techniques aiming to reduce the risk of obtaining results that are biased by unobserved heterogeneity. Colleagues [11] find for Jordan, for example, that women’s employment increases the risk of domestic violence (emotional, physical, and sexual) when endogeneity is not taken into account. By instrumenting women’s individual employment status with the cluster average of female employment (which can be interpreted as a proxy for local job opportunities for women), they find that the impact of women’s employment status on overall marital violence gets insignificant. When disaggregating different forms of domestic violence, the IV approach yields a weak evidence of a protective effect of women’s employment in the case of sexual violence. Similar to this cluster approach, colleagues [36] use geographic-level information on employment and unemployment for both men and women as instruments in their Spanish study. They find that, women’s employment significantly reduces their risk of experiencing domestic violence, but only when the partner himself is employed. Physical violence is found to be less common in more egalitarian couples (when both partners are employed) in Spain. Finally, colleagues [37] instrument women’s employment status by membership in a specific caste in India. With this approach, they yield evidence that women’s participation in paid work reduces domestic violence in India.

### Specific studies based on Turkey

Meta-analyses based on existing studies [38–40] identify low educated women and women who do not participate in the formal labor market as most at risk of experiencing domestic violence in Turkey. However, not all studies in the Turkish context deal with endogeneity issues and some of them are conducted on non-representative subsamples (female patients of one specific hospital, women of a given city, and students of certain universities …).

A study based on the 2008 wave of the nationally representative survey “National Research on Domestic Violence against Women in Turkey” (TDVAW), which verifies the level of collinearity between control variables and uses a complex sample matrix, finds that women who have no income or whose husbands contribute more to household income have a higher risk of experiencing domestic violence [41]. Yet, the same study finds no significant effect of female education or women’s overall employment on women’s risk of experiencing intimate partner violence.

Colleagues [4, 5] investigate the link between female employment and domestic violence in Turkey by using exogenous labor supply shocks as a robust methodological tool to control for endogeneity. They find that exogenous increases in female employment (due to an educational reform) lead to a higher risk of domestic violence, and exogenous declines in female employment (due to refugee inflows from Syria) reduce the risk of domestic violence in the context of Turkey. In their 2018 study [4], they use the 2008 TDVAW and address endogeneity by using the Turkish 1997 educational reform as a natural experiment. A regression-discontinuity design is applied between girls born before January 1987 and not concerned by the 1997 reform and girls born after January 1987 and concerned by the 1997 reform. They find that the increase in schooling years increased employment for rural women as well as the risk of experiencing economic control and psychological violence carried out by husbands. In their 2021 study [5], they use province-differences in terms of refugee inflows from Syria as exogenous labor supply shocks and find that refugee inflows decrease female employment as well as physical, sexual and psychological partner violence in the provinces that experienced high migrant inflows. The results of both studies suggest that domestic violence against women is caused by partners wanting to appropriate their wife’s income (instrument theory) and/or by male backlash in Turkey.

By exploiting both the 2008 and the 2014 wave of the TDVAW, colleague [6] investigates the correlation between female economic empowerment and domestic violence with the help of an Instrumental Variables procedure to reduce endogeneity. By using province averages of female employment as instrument for women’s employment status, [6] finds that once endogeneity is reduced, the positive correlation between female employment and exposure to domestic violence gets insignificant in Turkey.

What studies [4–6] have in common is that they do not explicitly distinguish between formal and informal working activities of women in Turkey throughout the analysis. They include social security status among the control variables, but do not always allow for ambiguous effects of formal and informal employment on domestic violence. However, in a rapidly transforming country like Turkey, women in formal and informal employment have very different socioeconomic backgrounds. Many women in informal jobs work have low educational backgrounds and work as contributing family workers from home, while formal jobs outside the house are still reserved to higher qualified women [42–44]. It is therefore possible that the exposure to domestic violence is highly polarized between formally and informally employed women in Turkey. In order to see whether formal employment outside the house comes hand in hand with a lower risk of experiencing domestic violence in Turkey in comparison to women who work in informal jobs and inactive women, we therefore treat formally and informally employed women in two distinct groups in our empirical analysis. In addition, we distinguish between women who do not work because the partner does not allow them to and women who are inactive for other reasons.

## Data source and measurement of domestic violence

Measures of domestic violence are found to be highly sensitive to context (including cultural differences and differences in reporting behaviors) and to methodology (community samples, nationally representative surveys, administrative data, etc.) [45–47]. Domestic violence is known to be among the most under-reported crimes for both men and women worldwide.

In this article, we use the Survey “National Research on Domestic Violence against Women in Turkey” (TDVAW)—a survey which was specifically designed to address the very sensitive topic of domestic violence. This concerns not only the data collection method, but also the information covered, as the Turkish Survey covers a wide set of variables that figure as potential determinants and consequences of domestic violence. Other, more general surveys like the Turkey Demographic and Health Survey (TDHS) or the Turkish Family Structure Survey (TAYA) are not specially designed to this end, which implies that they are likely to deliver biased measures of awareness and prevalence of domestic violence. In addition, these surveys cover a smaller range of variables that are potentially linked to domestic violence, and information is not always available for both women and their partners.

The Survey “National Research on Domestic Violence against Women in Turkey” was conducted for the first time in 2008, by the Hacettepe University Institute of Population Studies with support of TURKSTAT, financed by the Turkish Ministry of Family and Social Policies. The declared objectives of the survey are to identify the extent of violence against women, to determine the causes and to meet the need of data collection in this field. A second survey was carried out in 2014. Both the 2008 and the 2014 survey are cross-sectional.

The survey was conducted throughout Turkey using face-to-face interviews with women aged 15–59 and is representative at the national and regional level. The survey covers different forms of violence such as prevention of education or work in a paid job outside the house and disruption of daily life as a result of stalking, along with physical, sexual and emotional violence that women have experienced by their current or former intimate partners such as, their husbands, fiancés and boyfriends.

As the subject of this research is sensitive, the research was designed to ensure the safety of the interviewers conducting the fieldwork, while prioritizing the safety of respondents. A questionnaire developed by the World Health Organization (WHO) was taken as a model and was extended by adding particular subjects of interest for Turkey. Furthermore, The Ethical and Safety Guidelines established for violence against women studies by the WHO were followed in every stage of the research. The technical report of the Survey “National Research on Domestic Violence against Women in Turkey” can be found here: http://www.hips.hacettepe.edu.tr/ING_SUMMARY_REPORT_VAW_2014.pdf

The survey allows differentiating between controlling behavior, physical and sexual violence (lifetime and during the last 12 months). We focus our core analysis on *physical and/or sexual domestic violence against ever-married women aged 15–59*, *carried out by the (last) intimate male partner*, *during the last 12 months*. Our main dependent variable groups thus together physical and sexual violence in one binary variable. This allows focusing on violence that directly affects women’s physical integrity. Empirical results based on a more detailed distinction between different types of violence (only physical, only sexual, both physical and sexual, emotional, economic) can be found in the S1G Appendix. They point into the same direction as our results on physical and/or sexual violence.

The definitions of physical and sexual violence proposed in the Turkish Survey are as follows:

1. Physical violence against women by husband(s) or intimate partner(s): (a) slapped her or threw something at her that could hurt her; (b) pushed or shoved her or pulled her hair; (c) hit her with a fist or something else that could hurt her; (d) kicked, dragged her or beat her up; (e) choked or burned her; (f) threatened to use or actually used a gun, knife or other weapon against her.
2. Sexual violence against women by husband(s) or intimate partner(s): (a) physically forced her to have sexual intercourse; (b) had sexual intercourse when she did not want to because she was afraid of what partner might do; (c) forced her to do something sexual that she found degrading or humiliating.

The Survey “National Research on Domestic Violence against Women in Turkey” reports an overall prevalence rate of sexual and/or physical violence against ever-married women aged 15–59, carried out by the (last) intimate male partner during the last 12 months, of 13.7% for 2008 and 11% for 2014. The decline between the two years is relatively small and the prevalence rate of 2014 is still high in comparison to European standards (EU-28 average: 4%, as mentioned in section 2).

The empirical analysis of this article is based on the more recent 2014 survey. The 2014 survey has a target sample of 15,072 households. The response rate for women interviews is 83.3 percent. By selecting ever-married women aged 15–59, we obtain a sample of 6,125 women.

The distribution on physical and sexual violence within our dependent binary variable is the following: Among the 11% of ever-married women aged 15–49 in Turkey who have experienced physical and/or sexual violence by a partner in the last 12 months reported in the 2014 survey, somewhat more than one half (6.1 percentage points) has experienced only physical violence, while the other half is divided almost equally into women who have experienced only sexual violence (2.8 percentage points) and women who have experienced both physical and sexual violence (2.6 percentage points). The overlap is thus quite important and the prevalence rates for each of the three sub-groups (only physical/only sexual/both) are relatively small, which justifies that we do not distinguish between physical and sexual violence in our core analysis.

In addition, in order to maintain a sufficiently large variation of the endogenous variable within each category of the exogenous variables, our empirical analysis also does not distinguish between frequencies and types/severity of physical and/or sexual domestic violence. The Table in S1A Appendix shows that while there is an important diversity in terms of types of physical and sexual violence (intensity), most women have experienced such types repeatedly during the last 12 months. For example, among women who have experienced physical and/or sexual domestic violence during the last 12 months, 61.4% have been slapped, 45.9% have been shoved, 22.7% have been forced to have sexual intercourse and 7.3% have been threatened with a weapon. At the same time, for all nine reported types of violence, a large majority declares that the type of violence has happened more than twice. Sexual violence appears, however, to be more repeated than physical violence.

## Empirical specification

Our dependent variable is a binary variable which equals

- 1 if the woman has experienced physical and/or sexual domestic violence (at least one type), carried out by the (last) intimate male partner, during the last 12 months
- 0 otherwise.

Our target group is ever-married women aged 15–59.

To estimate women’s probability of experiencing physical and/or domestic violence, we apply a multivariate logistic regression model with robust standard errors.

The logistic regression model is preferred over a linear probability model (LPM), as the LPM is based on standard regression (estimated with OLS) and therefore bears the risk of predicting outcome probabilities outside the 0–1 range. Even when the LPM predicts values between 0 and 1 but close to either end of the interval (in our case close to 0 for some subgroups), the LPM may be mis-calibrated [48].

Our logistic regression model is a Generalized Linear Model (GLM) estimated with Maximum Likelihood. GLM is a flexible generalization of ordinary linear regression that allows for response variables that have error distribution models other than a normal distribution. By estimating a transformation of the probability rather than the probability itself, the logistic regression model always makes predictions between 0 and 1.

We estimate a woman’s probability of experiencing physical and/or sexual violence as a function of “woman’s economic empowerment”, a series of control variables capturing characteristics of the woman and her partner, and region fixed-effects.

The probability form of the multivariate logistic regression models woman’s probability of having experienced physical and/or sexual domestic violence (*π*) as:

$$\pi =\frac{e^{\beta_{o}+\beta_{1}*female\;econ.\;empowerment+\beta_{2}*controls+\beta_{3}*region}}{1+e^{\beta_{o}+\beta_{1}*female\;econ.\;empowerment+\beta_{2}*controls+\beta_{3}*region}}$$

with e = e constant or Euler’s number.

This transformation allows obtaining estimated coefficients rather than odds ratios, with a range from -*∞* to +*∞*. The estimated coefficients are, however, not marginal effects on probability, which makes the coefficients difficult to interpret in terms of magnitude. To facilitate their interpretation, we calculate predictive margins after the logistic estimations. Note that we use a probit rather than a logit model in order to be able to directly compare results of a simple probit model to results of a bi-probit model instrumenting endogenous regressors.

Our main independent variable of interest is “women’s economic empowerment”, which is measured in two ways:

1. by the woman’s participation in the formal labor market
2. by the woman’s contribution to household income.

We thus run two sets of specifications, one with women’s participation in the formal labor market as principal explanatory variable, and the other one with women’s contribution to household income as principal explanatory variable. We do not combine women’s labor market status and income contribution in one specification in the core analysis of this article in order to avoid excessive multicollinearity: Spearman’s rho correlation coefficients are relatively high, for example 0.45 between egalitarian income contribution and female formal employment (Pearson’s Chi-squared and Fisher’s exact tests: p = 0.000, indicating that the hypothesis of independence must be rejected).

Each set contains three regressions: We start with a categorical variable as principal explanatory variable. We then replace the categorical variable with a dummy variable in a second step, which enables us to replace the dummy variable by an instrument in a third step. The purpose of this procedure is to reduce endogeneity, as explained in further detail below.

When it comes to women’s employment status, we first distinguish between four categories: women who are in formal employment (employer, regular waged worker, regular salaried public official, regular self-employed), women in informal/irregular employment (seasonal or temporary daily wage worker, irregular self-employed, unpaid family worker), women whose partner does not allow them to work and women who are inactive for other reasons. We then contrast *formally* employed women to those who are not working or working in informal/irregular jobs. In this first set of regressions, we control for the partner’s labor market participation (formal and informal combined).

Women’s contribution to household income is first captured by five categories: she is the only income earner in the household, earns more than others, about the same as others, earns less than others, or earns nothing. In the questionnaire, women are asked about the money that they bring into the family. This refers primarily to income from labor, but also to other sources of regular monetary income (from savings, real estate, land etc.). Note that among the group of women who declare being contributors to household income, the large majority of women (80%) participate in the labor market (formal and informal activities combined). Within the group of women who declare contributing equally to household income, 80% of couples are both working. Within the group of couples in which no partner works, 88% of women declare contributing nothing to household income. 94% of women who do not work but their partner works declare contributing nothing to household income. Given the high but imperfect overlap between working status and income contribution, we combine working status and income contribution in regressions presented in the S1F Appendix. Note also that 63% of women who work on an informal and/or irregular basis declare contributing nothing to household income. This reinforces our claim that it is important to distinguish between informal and formal employment for women in the context of domestic violence in Turkey.

In the second step, we contrast women who contribute at least the same than others to the rest. Partner’s labor market participation is not controlled for in this set of regressions in our core analysis, as it is highly correlated with the partner’s contribution to household income, which is implicitly given by the measure of women’s contribution to household income. We do, however, control for partners who do not allow women to work as well as for the presence of other adult household members. In order to disentangle partnered women and those who are recently separated, we control for the current couple status (married and cohabiting vs. those whose partner recently died or who moved away due to separation or divorce). We do not restrict the sample to women who still live with their partners in order to avoid overlooking women who have recently separated due to domestic violence. Without the control for couple status, the estimated impact of a woman’s contribution to the household income on the risk of having experienced domestic violence in the last 12 months would be overestimated due to the fact that women who have recently separated from their husband because of domestic violence are observed as only (or mainly) contributors to household income.

We control in both sets for a range of demographic and social background characteristics for both women and their partners. These are: woman’s age, woman’s age difference with the partner, woman’s age at marriage, the type of marriage (free will, arranged with consent, arranged without consent), the number of marriages already experienced by the woman, the number of children still alive, the family type (nuclear or not), the partner’s mother tongue, the geographic area (rural or urban), the partner’s alcohol consumption, as well as the woman’s and her partner’s education level. For both women and their partners, we distinguish between three education categories: low (pre-primary and primary), middle (secondary) and high (tertiary). Additional ethnic background characteristics were also included in the regressions, but results are not presented here, as these characteristics all turned out to be insignificant (women’s mother tongue, type of place of residence before age 12, region lived before age 12). The same is valid for a blood connection with the husband.

All models include region fixed-effects (12 regions / Nuts-1). The considered region is the region the woman currently lives in (at the time of the survey). Region fixed-effects allow for a partial control of omitted variable bias. The economic empowerment of women is likely to be associated with characteristics that are sometimes difficult to measure (norms, values, socioeconomic context etc.), which may be associated with the quality of unions and the prevalence of domestic violence. These characteristics are not modeled explicitly, but they are controlled for as long as they are region-specific. Our fixed-effects models thus identify differences in the prevalence of domestic violence between socio-economic groups that exist *within* regions.

### Endogeneity concerns

Reverse causality between women’s labor market participation and income contribution on the one side and domestic violence on the other side is a key concern for our empirical analysis. The survey allows distinguishing between women whose partner or family does not allow them to work and those who are inactive for other reasons. This distinction is possible as the survey asks women for the reason for inactivity. The different reasons for inactivity suggested by the questionnaire are: housework, childcare, unemployed, job search, retired, student, disabled, sick, no need to work, not allowed to work, no education. As we found in previous work [12] that being forbidden to work is indicative of a controlling spouse, and controlling behavior often appears together with physical and/or sexual violence, this distinction makes it possible to treat women whose inactivity is likely to emerge as a consequence of being with a violent partner as a separate group in the regression analysis. This does, however, not completely rule out reverse causality between the remaining group of women (employed and inactive for other reasons) and domestic violence, as it remains possible that a woman chooses to be professionally active to be able to escape from her violent partner. It could also be that women who want to work have chosen partners who would not prevent them from working.

In order to increase the control for endogeneity caused by reverse causality between domestic violence and women’s employment status, respectively women’s income contribution, we apply an Instrumental Variable (IV) approach. We first replace the categorical employment and income contribution variables with binary ones (than can only take the values 0 or 1). For women’s labor market participation, we contrast formal employment against all other categories (unemployed, inactive, informally active, not allowed to work, other). For women’s income contribution, we contrast contributing ‘at least the same’ (all, more, about the same) than others to household income against all other categories (less, nothing). We then apply a bi-probit estimation (seemingly unrelated bivariate probit) implementing instrumental variables (IV). In order to be valid, our instruments have to be relevant (i.e. strongly correlated with women’s employment status, respectively women’s income contribution) and exogenous in the basic model (i.e. not correlated with women’s probability of experiencing physical and/or sexual domestic violence once women’s individual labor market status, respectively income contribution, is controlled for). There exist only a few candidates that have the potential to fulfill these conditions: current pregnancy, family size, recent separation, and regional averages of female formal employment and women’s income contribution. We found that the ‘cluster average of female formal employment’ is the best available valid instrument for women’s probability of being formally employed and that the ‘cluster average of women contributing at least the same as others to household income’ is the most valid instrument for women’s probability of contributing at least the same as others to household income. In surveys, clusters group together people of the same neighborhood. Clusters are not made for being representative for certain areas, but they are likely to cover individuals with similar socio-economic characteristics. These cluster averages thus proxy area-specific economic opportunities for woman, and eventually also existing normative barriers and network effects [11].. They can, however, not be considered as perfect instruments, as we cannot guarantee that the area averages affect women’s probability of domestic violence only through their impact on woman’s individual probability of being formally employed (respectively only through their impact on their probability of contributing at least the same to household income). It remains possible that the area-specific economic opportunities for women are affected by local gender norms, which also directly affect women’s risk of experiencing domestic violence (in that case, the exclusion restriction would be violated).

We confirm the validity of our instruments with several tests, which are presented in the S1 Appendix and discussed in the results section. The use of cluster averages as instruments does certainly not completely rule out endogeneity. They bear, however, the potential to reduce endogeneity, which is why we proceed with this approach. Our purpose is to see in how far our results change once we apply an IV-approach. If the empirical results appear to be sensitive to endogeneity, we have to put into question the results of the basic regression. This does, however, not mean that the IV-results are interpretable in terms of causality.

The geographical units that we use for calculating the cluster averages are census enumeration areas. In our sample, women are distributed over 517 clusters. The clusters contain between 1 and 24 observations, with a mean of 13 women per cluster. Following colleagues [11], we construct the instrumental variables in such a way that we use the cluster averages excluding the woman being considered in each observation. This allows us avoiding an in-built correlation.

We apply our IV-approach with a bivariate probit model estimated using full information maximum likelihood. Two binary dependent variables are estimated jointly here: The probability of being in formal employment (respectively of contributing at least the same as others to household income) as a function of the cluster average, and the probability of experiencing domestic violence as a function of the probability of being in formal employment (respectively of contributing at least the same as others to household income), while both estimations share the same list of control variables. The error terms of the two equations are allowed to be freely correlated in order to account for the possibility that some unobserved factors influence the two outcomes.

Finally, besides reverse causality between domestic violence and woman’s formal employment respectively her income to household contribution, endogenous controls could bias our estimation results. An ultimate step to address endogeneity consists therefore in dropping those control variables from the regression estimation which are potentially influenced by domestic violence, to see whether our results are robust. Endogeneity may concern in particular partner characteristics in terms of alcohol consumption and not allowing the woman to work (the partner might drink because he is violent and therefore ashamed; he might forbid the woman to work to hide obvious signs of domestic violence from the outside or to hinder her to leave). Endogeneity could also arise for several demographic characteristics of the couple, such as woman’s age at marriage, type of marriage, age difference with the partner, number of marriages, nuclear family and number of children. Finally, it cannot be completely excluded that the couple’s socio-economic characteristics in terms of partner’s labor market participation as well as of the woman’s and her partner’s level of education are endogenous.

## Descriptive statistics

Table 1 shows descriptive statistics. The first column shows the distribution (weighted proportion) of women across categories (full sample of ever married women aged 15–59: those who have experienced domestic violence and those who have not). The categories correspond to the regressors used in this study: employment status, education level, partner characteristics, demographic characteristics etc. The second column shows prevalence rates of physical and/or sexual domestic violence for each category (i.e. the weighted proportion of women among all women in each category who have experienced physical and/or sexual violence by the last husband during the last 12 month). We apply a Chi-squared test to verify if the prevalence rates are significantly different between categories. This is always the case except for family type (nuclear or not) and geographic area (rural/urban).

**Table 1:** Descriptive statistics.

	Distribution of women over categories	Proportion of women (among all women in each category) who have experienced phys. and/or sex. violence^1^
***All***	**1.00**	**0.11**
*Woman’s labor market status*		Chi^2^ = **
Formal employment	0.16	0.10
Informal/irregular employment	0.15	0.10
Partner/family does not allow working	0.07	0.20
Other inactive	0.63	0.10
*Partner’s labor market status*	1.00	Chi^2^ = **
Working	0.84	0.10
Not working	0.16	0.12
*Woman’s contribution to family income*		Chi^2^ = ***
Only she has income in the household	0.03	0.16
More than others	0.02	0.10
About the same than others	0.06	0.06
Less than others	0.14	0.11
Nothing	0.74	0.12
*Woman’s education*		Chi^2^ = ***
Low education (pre-primary, primary)	0.61	0.12
Middle education (secondary)	0.29	0.13
High education (tertiary)	0.11	0.08
*Partner education*		Chi^2^ = ***
Low education (pre-primary, primary)	0.43	0.13
Middle education (secondary)	0.41	0.12
High education (tertiary)	0.16	0.06
*Woman’s age*		Chi^2^ = ***
15–19	0.01	0.20
20–29	0.21	0.15
30–39	0.34	0.12
40–49	0.26	0.11
50–59	0.18	0.06
*Age difference with partner*		Chi^2^ = **
*>4 years*	0.46	0.11
*< = 4 years*	0.54	0.13
*Age at marriage*		Chi^2^ = ***
10–17	0.25	0.15
18–21	0.40	0.11
22–29	0.29	0.09
30+	0.06	0.09
*Type of marriage*		Chi^2^ = ***
Free will	0.43	0.10
Arranged with consent	0.48	0.11
Arranged without consent	0.09	0.22
*Number of children still alive*		Chi^2^ = **
0–2	0.63	0.11
3+	0.37	0.13
*Number of marriages*		Chi^2^ = ***
One	0.95	0.11
More than one	0.05	0.19
*Partner’s mother tongue*		Chi^2^ = ***
Turkish	0.83	0.11
Kurdish	0.13	0.14
Arabic	0.03	0.06
Other	0.01	0.05
*Nuclear family*		Chi^2^ = ns
*Yes*	0.44	0.11
*No*	0.56	0.11
*Alcohol consumption of the partner*		Chi^2^ = ***
Never	0.79	0.10
Every day/almost every day	0.03	0.28
Once or twice a week	0.04	0.19
A few times a month	0.05	0.17
Less than once a month	0.09	0.11
*Current couple status*		Chi^2^ = ***
Married	0.93	0.11
Partner recently died, separated, divorced	0.07	0.16
*Geographic area*		Chi^2^ = ns
Urban	0.78	0.12
Rural	0.22	0.11
*Region (Nuts-1)*		Chi^2^ = ***
Istanbul	0.21	0.11
West Marmara	0.04	0.10
Aegean	0.13	0.12
East Marmara	0.10	0.07
West Anatolia	0.10	0.14
Mediterrenian	0.13	0.11
Middle Anatolia	0.05	0.14
West Blacksea	0.06	0.10
East Blacksea	0.03	0.10
Northeast Anatolia	0.02	0.13
Middle East Anatolia	0.04	0.12
Southeastern Anatolia	0.09	0.15

^1^ Prevalence rates of domestic violence by last husband during last 12 month for each category.

Chi^2^ (Chi-squared test to verify if the prevalence rates are significantly different between categories): ns = not significant,

* p<0.05,

** p<0.01,

*** p<0.001

Data: Domestic Violence Survey Turkey, 2014; Sample: ever-married women aged 15 to 59.

When looking at the distribution of women across categories of employment status, Table 1 (first column, lines 2–5) shows that 16% of women in our sample work in formal employment, and 15% in informal/irregular employment. This suggests that the survey is representative in terms of female employment patterns in Turkey: In our sample, the female labor force participation rate is around 30%, similar to the rate reported by Turkstat Labor Force Survey, and there is a polarization between formal and informal/irregular employment. The Turkish Labor Force statistics report a female labor force participation rate for ages 15+ of 30.24% for the year 2014. In these official statistics, regular or casual employees, employers, self-employed as well as unpaid family workers are considered as ‘employed’, i.e. all economically active persons, be it formally or informally active (all people who supply labor for the production of goods and services during a specified period; see file:///C:/Users/70980/Downloads/Methodological%20note%20regarding%20the%20regulations%20made%20in%20the%20household%20%20labour%20force%20survey.pdf). In our sample, only ever-married women aged 15 to 59 are considered.

Table 1 shows furthermore that around 70% of ever married women aged 15–59 in our sample are not working. 7% are inactive because the partner and/or the family do not allow them to work, and 63% are inactive due to other reasons.

Table 1 shows that the overall prevalence of physical and/or sexual violence is 11% in our sample (first line, second column). Prevalence rates are, however, not the same for all types of labor market status (second column, lines 2–5): women who are not permitted to work are most exposed to domestic violence, while there is no difference in the prevalence rate between women who are inactive for other reasons, women who work in formal and women who work in informal employment. The high prevalence rate of domestic violence among women who are not allowed to work (20%) suggests that in particular for this group of women, domestic violence can figure as a determinant and not only as a consequence of their economic inactivity.

Table 1 shows furthermore that the large majority of partners in our sample is working, while woman with a partner who is not working have a somewhat higher prevalence rate of domestic violence.

74% of women in our sample contribute nothing to household income, and another 14% declare contributing less than others. Only 6% of ever-married women aged 15–59 in Turkey contribute about the same as other family members, only 5% of women contribute more than other family members, and only 3% are the only contributors. The prevalence of domestic violence differs much depending on women’s contribution to family income. The prevalence is largest for women at the two extremes, i.e. for those who contribute nothing (12%) as well as for those who are the only income earners (16%), while the prevalence rate is lowest for women contributing about the same than others (6%).

Women who have recently separated/divorced (or whose partner has recently died) represent 7% of women in our sample. Their prevalence rate of domestic violence (last 12 months) is much higher in comparison to women who still live with their married partner, which suggests that domestic violence was an issue for the separation. This could also contribute to the high prevalence rate of domestic violence of women who are the only contributors to household income.

Table 1 shows furthermore that the prevalence rate of physical and/or sexual violence is higher among young women and decreases with age. In 2014, 20% of ever-married women aged 15–19 declared having been subjected to physical and/or sexual violence by their husband or intimate partner during the last 12 months, while the prevalence is reduced by half for married women aged 40+. Also, the prevalence rate of domestic violence declines with women’s age at marriage. 15% of women who got married before the age of 18 are subject to domestic violence and women who have a partner who is more than 4 years older than them face a higher prevalence rate of violence than women with a younger partner.

In our sample, less than half of the women were able to choose freely their partner, but among the women whose marriage was arranged, the majority gave their consent. The prevalence rate of domestic violence is two times higher for women whose marriage was arranged without their consent (by family, eloped, abducted, bride exchange, etc.), in comparison to women who freely choose their partner and those who gave their consent to an arranged marriage.

The number of children seems positively linked to the prevalence of domestic violence. The prevalence rate also increases with the number of marriages. A very large majority (95%) of ever-married women in our sample are still in their first marriage, indicating that re-marriage after separation is not at all frequent in Turkey. Among remarried women, the prevalence rate of domestic violence (last 12 months) is almost twice as high in comparison to women who are married for the first time.

The descriptive statistics also show that the prevalence of domestic violence is somewhat higher among women whose partner’s mother tongue is Kurdish in comparison to partners whose mother tongue is Turkish, and much lower for partners of Arabic or any other mother tongue.

The difference in the prevalence of domestic violence between women living in a nuclear family (only the couple and their children) and women in other household configurations (mostly living with more family members) seems negligible.

What stands out, in contrast, are the differences according to the alcohol consumption of the partner. Those women whose partners never drink (or almost never) have a much lower prevalence of domestic violence than those who live with a partner who drinks on a regular basis. At the same time, a further descriptive analysis reveals that among those women in our sample who are exposed to domestic violence, the large majority have partners who never drink (70%; against 80% for women who are not exposed to domestic violence). This suggests that besides partners’ alcohol consumption, other determinants of domestic violence are important to consider.

Table 1 further shows that the majority of women in our sample are lower educated (61%), while middle educated women represent 29% and high educated women (with tertiary education) 11%. Men in Turkey are higher educated on average, with 16% of men having a university degree and 41% with secondary education. The prevalence of physical and/or sexual violence is lowest among women with tertiary education, while the difference between primary and secondary education seems negligible. The same is valid with regard to the partner’s level of education.

Finally, differences between rural and urban regions seem negligible. When comparing 12 Turkish regions (Nuts 1), the prevalence appears to be somewhat higher in the South-East and somewhat lower in the North-West.

## Regression results

### Women’s labor market participation

Table 2 shows regression results for our first set of specifications focusing on women’s labor market participation as exogenous variable.

**Table 2:** Estimated probability of having experienced sexual and/or physical violence by their husband/partner during the last 12 months. Core dependent variable: woman’s labor market status.

	Model 1 (probit)	Model 2 (probit)	Model 3 (bi-probit)
*Woman’s labor market status*			
Formal employment	*Ref*.	-0.0537	-1.183***
Informal/irregular employment	0.144*	*Ref*.	*Ref*.
Inactivity (other)	-0.0143		
Partner does not allow to work	0.338***		
*Partner’s labour market status*			
Working	*Ref*.	*Ref*.	*Ref*.
Not working	-0.0315	-0.0439	-0.0598
*Woman’s education*			
Low education (pre-primary, primary)	*Ref*.	*Ref*.	*Ref*.
Middle education (secondary)	0.106*	0.104*	0.183***
High education (tertiary)	-0.00933	-0.0188	0.503***
*Partner’s education*			
Low education (pre-primary, primary)	*Ref*.	*Ref*.	*Ref*.
Middle education (secondary)	-0.153***	-0.150***	-0.172***
High education (tertiary)	-0.401***	-0.415***	-0.373***
*Woman’s age*			
15–19	*Ref*.	*Ref*.	*Ref*.
20–29	-0.123	-0.137	-0.0488
30–39	-0.364*	-0.375*	-0.127
40–49	-0.598***	-0.605***	-0.335
50–59	-0.904***	-0.919***	-0.682***
*Age difference with partner*	-0.000542	-0.000650	0.000890
*Age at marriage*			
10–17	*Ref*.	*Ref*.	*Ref*.
18–21	-0.0744	-0.0847	-0.0959*
22–29	-0.0671	-0.0737	-0.0525
30+	-0.0955	-0.113	-0.101
*Type of marriage*			
Free will	*Ref*.	*Ref*.	*Ref*.
Arranged with consent	0.130**	0.133**	0.0572
Arranged without consent	0.486***	0.502***	0.441***
*Number of children still alive*	0.0656***	0.0602***	0.0322*
*Number of marriages*			
*One*	*Ref*.	*Ref*.	*Ref*.
*More than one*	0.343***	0.347***	0.313***
*Partner’s mother tongue*			
Turkish	*Ref*.	*Ref*.	*Ref*.
Kurdish	-0.0403	-0.0387	-0.0440
Arabic	-0.560***	-0.557***	-0.518***
Other	-0.181	-0.186	-0.179
*Nuclear family*	-0.0253	-0.0190	-0.0383
*Alcohol consumption of the partner*			
Never	*Ref*.	*Ref*.	*Ref*.
Every day/almost every day	0.687***	0.699***	0.743***
Once or twice a week	0.549***	0.559***	0.592***
A few times a month	0.404***	0.412***	0.496***
Less than once a month	0.195**	0.201**	0.293***
*Geographic area*			
Urban	*Ref*.	*Ref*.	*Ref*.
Rural	-0.0999*	0.347***	-0.0877*
*Region fixed effects (Nuts-1)*	*Yes*	*Yes*	*Yes*
Constant	-1.359***	-1.308**	-1.119***
N	6129	6129	6129
Pseudo R-sq	0.075	0.070	N/A

* p<0.10,

** p<0.05,

*** p<0.01.

Data: Domestic Violence Survey Turkey, 2014.

Sample: ever-married women aged 15 to 59.

All three models are estimated with robust standard errors and include region fixed effects (Nuts-1).

Model 1: simple probit model distinguishing between 4 different categories for woman’s working status (formal employment, informal/irregular employment, not allowed to work, inactivity for other reasons).

Model 2: simple probit model distinguishing between 2 different categories for woman’s working status (formal employment, any other).

Model 3: bi-probit model distinguishing between 2 different categories for woman’s working status (formal employment, any other.

Bi-probit: seemingly unrelated bivariate probit implementing instrumental variables.

The endogenous regressor ‘woman’s formal employment’ is instrumented with the cluster average (= proportion of women in formal employment in each cluster (cluster = census enumeration area). The cluster excludes the woman being considered in each observation to avoid in-built correlation. Two binary dependent variables are estimated jointly: The probability of being in formal employment as a function of the cluster average, and the probability of experiencing domestic violence as a function of the probability of being in formal employment, while both estimations share the same list of control variables. The error terms of the two equations are allowed to be freely correlated in order to account for the possibility that some unobserved factors influence the two outcomes. Results of the model with formal employment as dependent variable can be found in the Table in S1B Appendix, model A.

Model 1 tests the correlation between woman’s labor market status and the probability of experiencing domestic violence by distinguishing between formal employment, informal/irregular employment, not being allowed to work and inactivity due to other reasons. Model 1 shows that women who are not allowed to work have the highest probability of experiencing domestic violence, confirming that limited freedom in terms of economic participation and domestic violence are closely related events. Moreover, and most importantly, the results of Model 1 show that women in informal/irregular employment have a significantly higher probability of experiencing domestic violence than women in formal employment as well as women who are inactive due to other reasons (statistical significance at the 10% level). The difference in exposure to domestic violence between formally employed women and women who are inactive (due to other reasons than not being allowed to work) is insignificant.

**Fig 1:**
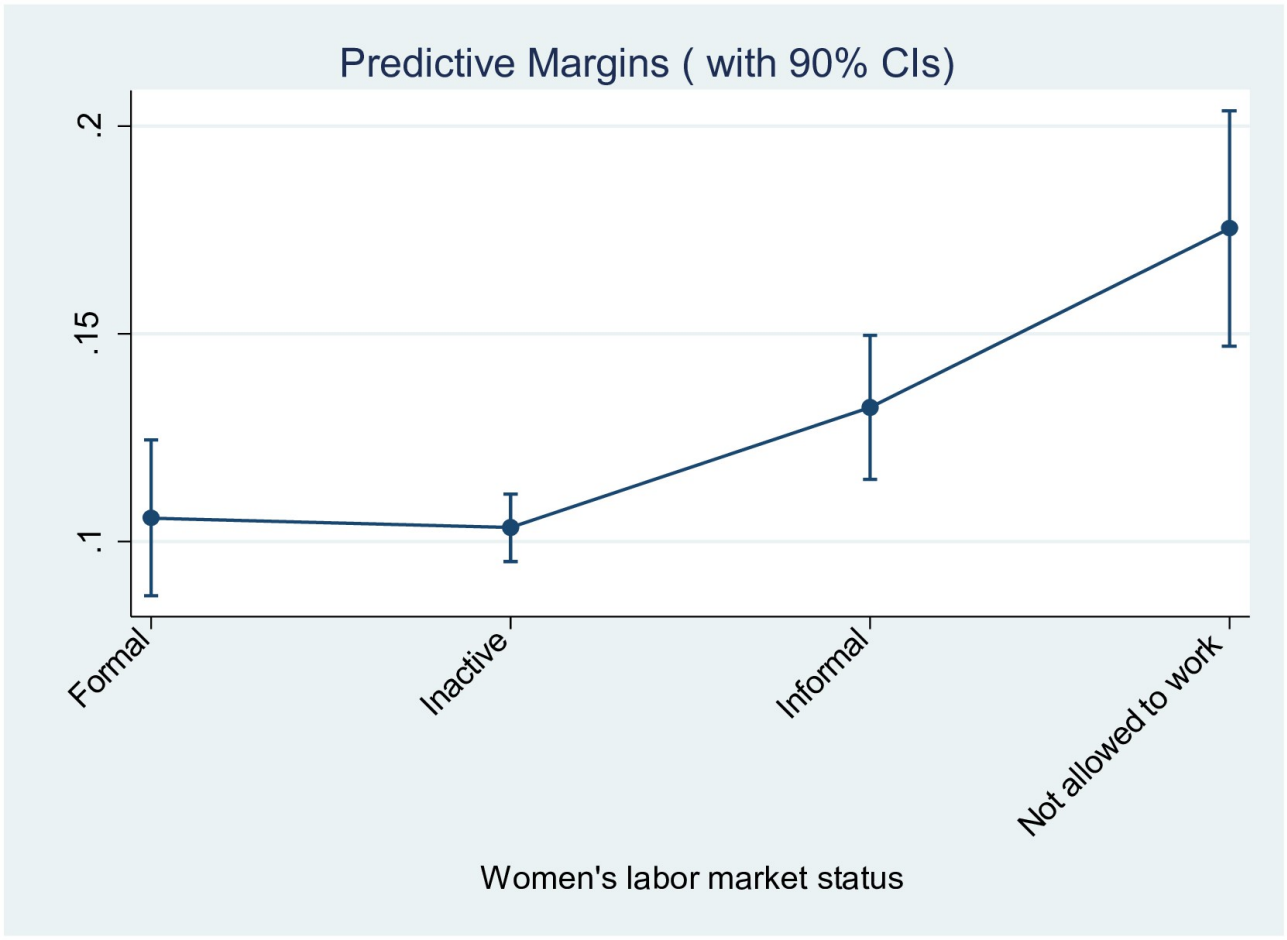
Data: Domestic Violence Survey Turkey, 2014. Sample: ever-married women aged 15 to 59.

Fig 1 illustrates the probabilities of experiencing domestic violence predicted by model 1 for the four categories (predictive margins at means). The predictive margins are 13.2% for informally employed women, 10.6% for formally employed women, 10.3% for women who are inactive (due to other reasons than not being allowed to work) and 17.5% for women who are not allowed to work. Note that confidence intervals can overlap in the Figure even though differences between categories are statistically significant.

When we consider female employment as a whole and distinguish only between women who work (formal, informal and irregular employment combined) and those who are inactive (while controlling for ‘not being allowed to work’), we find that working women have a significantly higher probability of experiencing domestic violence than inactive women (regression results presented in the Table in S1B Appendix). This finding is in line with other recent empirical studies on Turkey which suggest that working women are more exposed to domestic violence than non-working women [4–6].

Our distinction between formal and informal work reveals, however, that only those women working in informal jobs are more exposed to domestic violence than non-working women, while women in formal jobs do not have higher prevalence rates than non-working women. This suggests that distinguishing between formal and informal employment is important when analyzing patterns of domestic violence in Turkey.

The finding that women in formal employment are significantly less exposed to domestic violence than women in informal working activities is likely to be due to women in formal and informal employment having very different socioeconomic backgrounds in Turkey. Many women in informal/irregular employment work as contributing family workers (i.e. they work at home), while women in formal employment tend to occupy white-collar jobs in the service sector, i.e. outside home, which require a certain level of qualification [42–44, 49–51]. Female employment is very polarized between these two categories in Turkey: about half of the professionally active women in our sample work in formal employment, while the other half are engaged in informal and irregular working activities (see Table 1). Inactive women belong to an “intermediate social category” in Turkey (once we separate women whose partners and/or the family do not allow them to work from those who are inactive due to other reasons). Most partnered women who ‘have chosen to be’ inactive in Turkey are likely to belong to a group of higher social status in comparison to partnered women who work in informal/irregular employment, as the literature on the evolution of female employment during times of economic and social change suggests (see [50] for an overview): partners of inactive women can ‘afford’ that their wife does not work (a ‘middle class’ phenomenon based on the male breadwinner model), because the husband’s earnings are considered as sufficient to maintain the family. Besides, according to colleague [50], sending a married woman to work in a blue-collar job would stigmatize the man (because it suggests that the husband does not earn much). Once a woman gets more educated, however, she can access a white-collar job, and the stigmatization effect vanishes. Consequently, lower and middle educated women are the most likely to participate in the labor market, while inactivity is highest among middle educated women (the ‘feminization U’). The educational advantage enables higher educated women to participate in the service sector, while women with lower education often engage in agricultural activities, which are frequently informal/irregular. The affiliation to higher “social categories” might explain our finding that women in formal employment as well as inactive women are less prone to experience domestic violence than women who work in informal/irregular activities.

However, we cannot exclude that the results of Model 1 suffer from endogeneity. In order to prepare an IV approach, Model 2 groups together women who are inactive and who work in informal/irregular jobs and contrasts them to women who are in formal employment. In this simple setting, no significant difference is found between the two groups The predictive margins are 11.6% for women who are inactive or who work in informal/irregular jobs and 10.6% for women in formal employment. The interpretation of the estimatedcoefficients remains problematic due to potential inverse causality between domestic violence and women’s participation in the formal labor force. If domestic violence causes women to seek work outside the house to escape from their partners, the protective role of formal employment risks being underestimated in Model 2. If violent partners hinder women to work in the formal sector (outside the house), the protective role of formal employment risks being overestimated.

We thus apply a bi-probit approach instrumenting women’s formal employment by the cluster average. Model 3 indicates that once we increase our control for endogeneity, the coefficient of female formal employment gets more negative and highly significant: Women in formal employment are now found to have a significantly lower probability of experiencing domestic violence in comparison to the counter group of women who are not participating in the formal labor market. It is thus likely that the correlation between female formal employment and domestic violence is insignificant in the simple probit model (Table 2, Model 2) due to reverse causality caused by women seeking formal employment to escape from domestic violence. However, as cluster averages are likely to be imperfect instruments, endogeneity cannot be completely ruled out, which is why we abstain from making strong causal interference.

To evaluate the validity of our instrument, we now test if the instrument is strongly correlated with the endogenous variable (relevance of the instrument) and exogenous in the basic model (exclusion restriction). The probability of working in a formal job has been estimated simultaneously by the bi-probit model, which includes the cluster average among the repressors. The estimation result can be found in the Table in S1C Appendix, model A. It shows that the cluster average of female formal employment is highly significant and positively correlated with women’s probability of formal employment. The strong correlation between the instrument and the endogenous regressor confirms the relevance, or predictive power, of our instrument.

To test if our instrument is exogenous (exclusion restriction), we now estimate the correlation between the cluster average and domestic violence. Regression results can be found in the Table in S1D Appendix, model C. The cluster average of female formal employment is found to be not directly correlated with women’s probability of experiencing domestic violence, once the instrument is included in the regression along with the individual observation of a woman’s labor market status and our series of control variables. The cluster average thus affects domestic violence only through women’s formal employment. With the inclusion of a set of controls for the woman’s and her partner’s characteristics, it seems that the instrument reflects local employment conditions for women as well as local attitudes toward women’s employment that do not directly influence domestic violence.

We also applied formal tests obtained from two-stage linear probability models and IV-probit models with continuous endogenous variables. These tests (Wald test of exogeneity, Cragg-Donald Wald F statistic based on [52]: weak identification test, underidentification test based on [53], weak instrument robust test) suggest that our instruments are valid and strong. However, these test results are not to 100% reliable, as linear models are less appropriate for our data than probit and bi-probit models. By focusing on bi-probit models when applying our IV-approach, we follow the recommendations of colleagues [48], who have tested against each other linear IV and bivariate probit models with an endogenous binary treatment and binary outcome. They find that the linear IV estimates exhibit larger coefficients and standard errors and that bi-probit performs especially well when the treatment probability is close to 0 or 1. In our sample, the proportion of women having experienced domestic violence is smaller than 10% for several categories of women (see Table 1). In this case, the confidence intervals of the estimated coefficients of linear IV models remain too large according to [48], even with large sample sizes.

Based on our set of regressions and tests, we conclude that estimations of the association between female formal employment and domestic violence do not yield consistent results without efforts to reduce reverse causality between the two variables, that the cluster average of female formal employment can be considered as a valid instrument for the formal work status of women, and that the correlation between domestic violence and female formal employment is significantly negative once reverse causality between the two variables is reduced.

Finally, an ultimate step to address endogeneity in this setting consists in dropping those control variables which are potentially influenced by domestic violence, to see whether results are robust. We therefore re-estimate the bi-probit model (as well as Models 1 and 2) without controls for the partner’s alcohol consumption and for ‘not allowed to work’. In that case, women who are not allowed to work are grouped together with women who are inactive for other reasons. Results are shown in the Table in S1E Appendix and are similar to results shown in Table 2. Notably, the bi-probit model (Model G) yields a significantly negative association between woman’s formal employment and domestic violence.

We further tested the robustness of our results by dropping potentially endogenous demographic characteristics of the couple (woman’s age at marriage, type of marriage, age difference with the partner, number of marriages, nuclear family and number of children) in a second step. In a third step, we dropped partner’s labor market status as well as the woman’s and her partners level of education. We did not drop all the above-mentioned control variables simultaneously in order to preserve the explicative power of the model. In all cases, the bi-probit model yielded a significantly negative association between woman’s formal employment and domestic violence. The regional clusters stayed valid and strong instruments.

### Woman’s contribution to household income

Table 3 shows regression results for our second set of specifications focusing on woman’s contribution to household income as exogenous variable. All three specifications contain controls for demographic characteristics of the woman and her partner, partner’s mother tongue, partner’s alcohol consumption, the woman’s and her partner’s level of education, the geographic area and region fixed effects. We also control for partners who do not allow their wives to participate in working activities outside the house/family. We further include the current couple status in order to capture, among the main income contributors, those women who have recently separated from their husbands because of domestic violence.

**Table 3:** Estimated probability of having experienced sexual and/or physical violence by their husband/partner during the last 12 months. Core dependent variable: woman’s contribution to household income.

	Model 4 (probit)	Model 5 (probit)	Model 6 (bi-probit)
*Woman’s contribution to family income*			
About the same than others	*Ref*.	-0.0861	-0.488**
Only she has income in the household	0.417**		
More than others	0.165		
Less than others	0.243*	*Ref*.	*Ref*.
Nothing	0.241**		
*Partner/family does not allow to work*	0.324***	0.326***	0.289***
*Woman’s education*			
Low education (pre-primary, primary)	*Ref*.	*Ref*.	*Ref*.
Middle education (secondary)	0.0995*	0.0989*	0.111*
High education (tertiary)	0.00577	-0.00655	0.111
*Partner’s education*			
Low education (pre-primary, primary)	*Ref*.	*Ref*.	*Ref*.
Middle education (secondary)	-0.150***	-0.149***	-0.154***
High education (tertiary)	-0.393***	-0.398***	-0.392***
*Woman’s age*			
15–19	*Ref*.	*Ref*.	*Ref*.
20–29	-0.128	-0.131	-0.111
30–39	-0.369*	-0.371*	-0.328
40–49	-0.602***	-0.603***	-0.549***
50–59	-0.923***	-0.925***	-0.869***
*Age difference with partner*	-0.00175	-0.00173	-0.00138
*Age at marriage*			
10–17	*Ref*.	*Ref*.	*Ref*.
18–21	-0.0812	-0.0771	-0.0824
22–29	-0.0731	-0.0725	-0.0744
30+	-0.118	-0.115	-0.119
*Type of marriage*			
Free will	*Ref*.	*Ref*.	*Ref*.
Arranged with consent	0.131**	0.134**	0.126**
Arranged without consent	0.498***	0.493***	0.487***
*Number of children still alive*	0.0651***	0.0662***	0.0594***
*Number of marriages*			
*One*	*Ref*.	*Ref*.	*Ref*.
*More than one*	0.343***	0.346***	0.329***
*Partner’s mother tongue*			
Turkish	*Ref*.	*Ref*.	*Ref*.
Kurdish	-0.0486	-0.0474	-0.0445
Arabic	-0.576***	-0.575***	-0.573***
Other	-0.172	-0.177	-0.168
*Nuclear family*	0.00152	0.00098	-0.00244
*Alcohol consumption of the partner*			
Never	*Ref*.	*Ref*.	*Ref*.
Every day/almost every day	0.649***	0.649***	0.670***
Once or twice a week	0.533***	*0*.524***	0.525***
A few times a month	0.413***	0.407***	0.420***
Less than once a month	0.208**	0.200**	0.221***
*Current couple status*			
Married	*Ref*.	*Ref*.	*Ref*.
Widow, separated, divorced (recently)	0.115	0.197**	0.343***
*Geographic area*			
Urban	*Ref*.	*Ref*.	*Ref*.
Rural	-0.0726	-0.0709	-0.0807
*Region fixed effects (Nuts-1)*	*Yes*	*Yes*	*Yes*
Constant	-1.594***	-1.366***	-1.331***
N	6129	6129	6129
Pseudo R-sq	0.076	0.075	N/A

* p<0.10,

** p<0.05,

*** p<0.01.

Data: Domestic Violence Survey Turkey, 2014.

Sample: ever-married women aged 15 to 59.

All three models are estimated with robust standard errors and include region fixed effects (Nuts-1).

Model 1: simple probit model distinguishing between 5 different categories for woman’s contribution to household income: about the same, all, more, less, nothing.

Model 2: simple probit model distinguishing between 2 different categories for woman’s contribution to household income: at least the same (combining about the same, all and more) against less or nothing (combined).

Model 3: bi-probit model distinguishing between 2 different categories for woman’s contribution to household income: at least the same (combining about the same, all and more) against less or nothing (combined).

Bi-probit: seemingly unrelated bivariate probit implementing instrumental variables.

The endogenous regressor ‘woman contributing at least the same’ is instrumented with the cluster average (= proportion of women contributing at least the same in each cluster (cluster = census enumeration area). The cluster excludes the woman being considered in each observation to avoid in-built correlation. Two binary dependent variables are estimated jointly: The probability of ‘contributing at least the same’ as a function of the cluster average, and the probability of experiencing domestic violence as a function of the probability of ‘contributing at least the same’, while both estimations share the same list of control variables. The error terms of the two equations are allowed to be freely correlated in order to account for the possibility that some unobserved factors influence the two outcomes. Results of the model with ‘contributing at least the same’ as dependent variable can be found in the Table in S1B Appendix, model B.

Model 4 shows that women who contribute about the same as other family members to household income (our reference category) have the lowest risk of experiencing domestic violence. Compared to them, women who are at the two extremes of the within-household income distribution (i.e. women who contribute all or nothing to household income) suffer a significantly higher risk of domestic violence. This is also the case for women who contribute less than others to household income. It should be noted that we do not only control for women who have recently separated, but also for those who do not live in nuclear families. This implies that the coefficients of women’s contribution to family income can be interpreted as follows: among women who live with their husbands (i.e. without any further adult household members), those who contribute less than others (i.e. their partners) or nothing to household income, and those who are the only income contributors, have a significantly higher risk of experiencing domestic violence than those who earn about the same or more than others (i.e. their partners).

**Fig 2:**
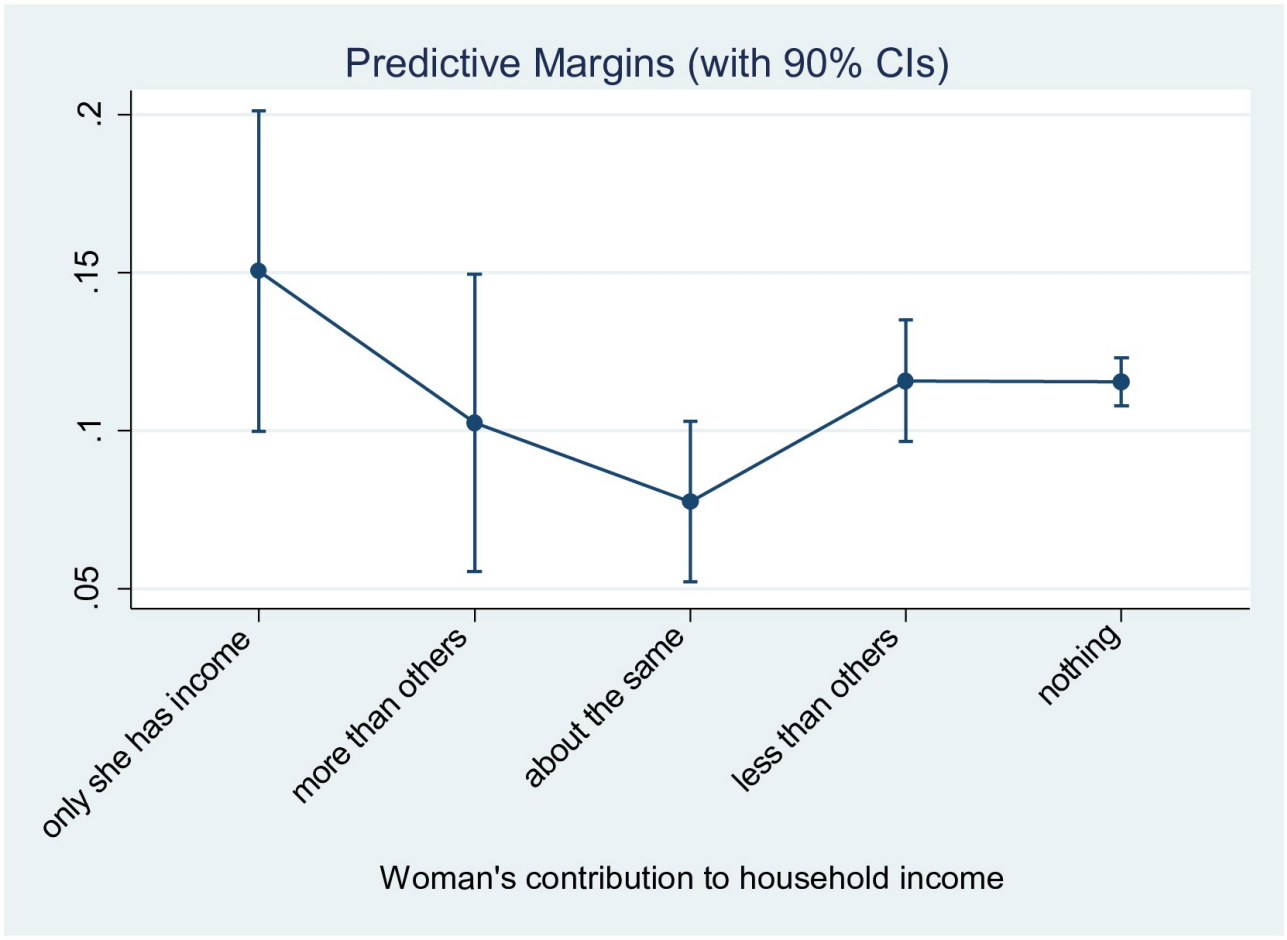
Data: Domestic Violence Survey Turkey, 2014. Sample: ever-married women aged 15 to 59.

Fig 2 illustrates the predictive margins (at means) of experiencing domestic violence predicted by model 4 for the five categories of female contribution to household income.

Fig 2 shows that women who are the only contributors to family income have, with 15%, the highest predictive margins of experiencing physical and/or sexual domestic violence. In our sample, partnered women who are the only income contributors are more likely to have a partner whose economic inactivity is unintentional (unemployed, injured and disabled) than the average. It is possible that the unequal distribution of economic resources creates tensions within the couple, which can result in domestic violence (backlash effect). However, it is also possible that women who are with a violent (and professionally inactive) partner tend to work outside the house in order to escape from violence. On the other hand, Fig 2 shows that contributing nothing or less to household income is also strongly correlated with domestic violence (predictive margins: 12%). Those with the least exposure to domestic violence are women who contribute equally to household income (7%). Endogeneity can, however, affect these results, as violent partners might more than others hinder women from work (at least from work outside the house).

In order to prepare our IV approach, Model 5 groups together women who contribute ‘at least the same’ as other household members (i.e. all, more, and about the same) to household income and contrasts them to women who contribute less or nothing to household income. In this simple probit regression, the estimated coefficient of the category ‘‘at least the same’ is negative, but rather small and insignificant. The predictive margins are 10% for women who contribute at least the same and 11.6% for women who contribute less or nothing than other household members.

To reduce endogeneity, we now apply a bi-probit approach instrumenting women’s contribution to household income by the cluster average (proportion of women who contribute at least the same to household income as other household members). Model 6 indicates that the estimated coefficient of ‘contributing at least the same’ gets more negative and increases in significance once we apply our bi-probit approach. With an increased control for endogeneity, women who contribute at least the same to household have a significantly lower probability of experiencing domestic violence in comparison to women who contribute less or nothing to household income.

To evaluate the validity of our instrument, we now test again the relevance of the instrument and the exclusion restriction. Woman’s probability of contributing at least the same as others to household income has been estimated simultaneously by the bi-probit model. The estimation result can be found in the Table in S1C Appendix, Model B. The correlation between the cluster average and women’s probability of contributing at least the same as others to household income is significantly positive, suggesting that the instrument has predictive power. Model D in the Table in S1D Appendix further shows that the cluster average of women ‘contributing at least the same to household income’ is found to be not directly correlated with women’s probability of experiencing domestic violence, once the instrument is included in the regression along with the individual observation of a woman’s labor market status and our series of control variables. This confirms the exclusion restriction.

We conclude thus that cluster average of ‘women contributing at least the same than others to household income’ can be considered as a valid instrument for women’s contribution to household income, and that women who earn at least the same as other household members have a significantly reduced risk in experiencing physical and/or sexual domestic violence in comparison to those who earn less or nothing once endogeneity is reduced. When potential endogenous regressors are dropped from the regressions (partner’s alcohol consumption & ‘not allowed to work’; woman’s age at marriage & type of marriage & age difference with the partner & number of marriages & nuclear family & number of children; woman’s and her partners level of education), the bi-probit models confirm a significantly negative association between women contributing at least the same to household income and domestic violence.

We now add the couple’s employment status (formal and informal combined) to the set of regressors in Models 4, 5 and 6. This allows us to see if the association between women’s contribution to household income and domestic violence holds independent of employment.

We distinguish between ‘neither work’ (12.5% of couples in our sample), ‘only he works’ (57.1%), ‘both work’ (26.9%), and ‘only she works’ (3.5%). Regression results can be found in the Table in S1F Appendix. Model H shows that women who contribute about the same as others to household income are confirmed to have a significantly lower risk of experiencing domestic violence in comparison to women who contribute less than others or nothing, independent of the couple’s employment configuration. Model I shows that controlling for the couple’s employment configuration reinforces the negative association between women ‘contributing at least the same than others to household income’ and domestic violence. The estimated coefficient is now significant even in the simple probit model. Model J confirms that our IV-approach reinforces the negative association between women ‘contributing at least the same than others to household income’ and domestic violence. When we ad female employment (formal and informal combined) and partner employment as two separate control variables in the bi-probit regression, the estimated coefficient of ‘women contributing at least the same than others to household income’ is negative, but not significant anymore. However, the negative coefficient of ‘at least as much’ is significant when we control for female employment only, when we control for partner employment only as well as when we control for female formal employment only (while female formal employment is insignificant). All instruments stay valid in the bi-probit settings which combine contribution to household income and employment.

Due to the high collinearity between employment and contribution to household income mentioned in section 4, the interpretation of the estimated coefficients is however quite problematic. We consider thus that only regression results of Table 2 should be used when it comes to making substantial statements about the association between women’s formal employment and domestic violence, and only regression results of Table 3 should be used when it comes to making substantial statements about the association between women’s contribution to household income and domestic violence.

### Results for different types of domestic violence

We now apply a robustness check for our basic regression results. More specifically, we want to see if the basic results of our analysis, i.e. lower incidence rates for formally employed women (Model 1) and for women who contribute equally to household income (Model 4) hold when distinguishing in more detail between different forms of domestic violence. We therefore proceed with separate regressions based on subgroups of domestic violence. Results are shown in the Table in S1G Appendix. We distinguish here between five different types of domestic violence: only physical, only sexual, both physical and sexual, emotional and economic. Emotional violence/abuse against women by last husband or intimate partner over the last 12 months is defined as follows (at least one): Insulted her or swore at her; belittled or humiliated her in front of other people; scared or threatened her; threatened to hurt her or someone that she cared about. Economic violence/abuse against women by last husband or intimate partner over the last 12 months is defined as follows (at least one): Caused her to quit her job; did not give her money for household expenses; deprived her of her income.

In our sample, 26% of women report to have experienced emotional, and 15% of women report to have experienced economic violence by the partner during the last 12 months. Prevalence rates for sub-categories of physical and/or sexual domestic violence are: 6.1% only physical, 2.8% only sexual and 2.6% both. Note that information on different types of controlling behavior (isolation from friends and family, intervention with clothes etc.) is only available on a lifetime basis in the survey.

The first five columns present results with women’s labor market status, and the last five columns present results with women’s income contribution as main exogenous variable of interest. Regressions on the probability of sexual domestic violence (columns 2 and 5) show a somewhat reduced sample size (due to zero reported variation of this specific endogenous variable within the group ‘Partner’s mother tongue: Arabic’). The estimated confidence intervals are quite high for ‘only sexual’, ‘only physical’, and ‘both sexual and physical’, probably due to combinations of categories that lead to rare events. The low significance does therefore not necessarily urge us to reject the tested hypotheses. Overall, the estimated coefficients for female formal employment and women’s income contribution point in the same direction as results of Models 1 (Table 2) and 4 (Table 3). For all types of domestic violence -be it physical, sexual, emotional or economic- formally employed women are found to have a reduced risk in comparison to informally employed women, and women who earn about the same as their partners have a reduced risk in comparison to those who earn less or nothing.

## Conclusion

This article shows that the relation between women’s economic empowerment and spousal violence in Turkey is complex and interrelated. By using data from the Survey “National Research on Domestic Violence against Women in Turkey” conducted in 2014, we find that female employment -per se- cannot be associated with lower incidence rates of physical and/or sexual domestic violence in Turkey. However, our analysis suggests that women who work in the *forma*l labor market as well as women who gain at least as much as their partners have a lower risk of experiencing physical and/or sexual domestic violence in Turkey.

This clear-cut finding has been obtained due to two important methodological approaches: First, women’s employment status has been observed by distinguishing between four categories: Formal employment (outside the house), informal/irregular employment, inactivity due to limited freedom of movement/economic participation, and inactivity due to other reasons. This distinction makes it possible to treat women whose inactivity is likely to emerge as a consequence of being with a violent partner as a separate group in the regression analysis. Furthermore, the distinction between women who are formally employed and women who work in informal activities allows taking into account that women in formal and informal employment have very different socioeconomic backgrounds in a country like Turkey, which currently undergoes important economic and social transformations. Many women in informal employment work as contributing family workers from home, which goes with a relatively low economic status within the family. The large majority of women working in informal employment in our sample considers that they do not contribute to family income. Women in formal employment tend to occupy white-collar jobs in the service sector, i.e. outside home, which often require a higher level of education. These jobs mostly provide women with an independent payment. Inactive women seem to represent an “intermediate social category” in Turkey. It is therefore problematic to not explicitly distinguish between formally and informally employed women when analyzing patterns of domestic violence in Turkey.

Second, cluster averages have been used to instrument female formal employment and women’s income contribution with the intention to reduce endogeneity. Albeit these cluster averages cannot be considered as perfect instruments, our bi-probit regression results suggest that without this control, the protective role of women’s formal employment and women’s contribution to household income would be underestimated. This underestimation is likely to emerge due to inverse causality caused by women who work outside home in order to escape from domestic violence. Once this reverse causality is reduced (albeit not eliminated), we find that: (1) formally employed women have a significantly lower risk of experiencing physical and/or sexual domestic violence in comparison to those who are informally employed and inactive, and (2) women who contribute at least the same as others to household income have a significantly lower risk of experiencing physical and/or sexual domestic violence in comparison to those who contribute less or nothing to household income.

As our cluster averages are not perfect instruments, our study does not intend to challenge the previous, more methodologically robust studies on Turkey proposed by colleagues [4 and 5], who find that exogenous increases in female employment lead to a higher risk of domestic violence, and exogenous declines in female employment reduce the risk of domestic violence in the context of Turkey. Rather, our study points to the usefulness of explicitly distinguishing between formal and informal female employment, all by confirming that estimation results are sensitive to controls for endogeneity.

Our finding that women in formal jobs are less exposed to physical and/or sexual domestic violence than women in informal jobs in Turkey leaves room for the possibility that the theoretical predictions of bargaining models of domestic violence also apply to the Turkish context: Paid work in the formal sector outside the household may improve women’s decision-making power inside the household and increase their capacity to threaten divorce, which can reduce their exposure to domestic violence [15–19]. Besides, as women working in the informal sector are likely to spend more time with their intimate partners than women working in the formal sectors (in particular daily and seasonal workers and those working in subsistence activities), they might be more exposed to their partners during the day. Besides bargaining power, exposure duration is thus another mechanism that could explain why women in formal employment are less exposed to domestic violence than women in informal jobs in Turkey.

In the light of our findings, policy measures which encourage higher education and formal employment for women are relevant in Turkey. There still exist important gender gaps in terms of education, participation in the formal labor market and earning opportunities in Turkey. Many girls, especially in the rural areas, are still forced to marry early, which limits their chances of access to better education, and the majority of ever-married women in Turkey are in an arranged marriage. Lower educational attainment leads to less employability in the formal sector and therewith to economic dependence from the partner. Today, gender differences in terms of access to higher education still exist to some extent in Turkey. In 2019, in Turkey 76% of men aged 25+ had at least completed lower secondary education in Turkey, versus 56% of women (World Bank World Development Indicators). Only about one third of Turkish women participate in the formal labor market. The female labor force participation rate (% of female population ages 15+) in Turkey was 30.2% in 2014 and has only increased slightly since then, to 33.5% in 2019 (modelled ILO estimate: International Labour Organization, ILOSTAT & World Bank World Development Indicators, as well as Turkstat Labor Force Suvey). Most women in Turkey still stop working once they have children [54]. Higher education and formal wage employment would allow Turkish women not only to gain economic independence, but also to choose their partner freely.

Our case study for Turkey suggests that disentangling between formal and informal female employment can be a useful tool for future empirical studies on domestic violence in countries which undergo rapid socio-economic transformations. Moreover, our study points to the necessity of further data collection on domestic violence in Turkey. Due to the cross-sectional setting of the Survey “National Research on Domestic Violence against Women in Turkey”, only imperfect controls for endogeneity can be applied. Only a longitudinal survey design would enable researchers to holistically investigate the causal mechanism that can lead to -and that can facilitate women to escape from- domestic violence in Turkey.

## Supporting information

S1 Appendix (DOCX)
