# Supplementary material for: The association between women’s economic participation and physical and/or sexual domestic violence against women: A case study for Turkey
Source: PLoS One. 2022 Nov 16;17(11):e0273440. doi: 10.1371/journal.pone.0273440 (PMC9668192; doi:10.1371/journal.pone.0273440)
Supplement: S1 Appendix — (DOCX) [file pone.0273440.s001.docx]

# Appendix

*Appendix A: Types and frequency of sexual and/or physical domestic violence*

Data: Domestic Violence Survey Turkey, 2014

*Appendix B: Estimated probability of having experienced sexual and/or physical violence by their husband/partner during the last 12 months (simple probit)*

*Core dependent variables: Woman’s labor market status*

| *Woman's labor market status* | |  |
| --- | --- | --- |
|  | Working (formal & informal employment) | *Ref.* |
|  | Inactivity (other) | -0.101* |
|  | Partner does not allow to work | 0.252*** |
| *Partner's labour market status* | |  |
|  | Working | *Ref.* |
|  | Not working | -0.036 |
| *Woman's education* | |  |
|  | Low education (pre-primary, primary) | *Ref.* |
|  | Middle education (secondary) | 0.099 |
|  | High education (tertiary) | -0.047 |
| *Partner's education* | |  |
|  | Low education (pre-primary, primary) | *Ref.* |
|  | Middle education (secondary) | -0.153*** |
|  | High education (tertiary) | -0.405*** |
| *Woman's age* | |  |
|  | 15-19 | *Ref.* |
|  | 20-29 | -0.125 |
|  | 30-39 | -0.371* |
|  | 40-49 | -0.603*** |
|  | 50-59 | -0.906*** |
| *Age difference with partner* | | -0.001 |
| *Age at marriage* | |  |
|  | 10-17 | *Ref.* |
|  | 18-21 | -0.075 |
|  | 22-29 | -0.071 |
|  | 30+ | -0.101 |

*…*

*Appendix B continued*

| *Type of marriage* | |  |
| --- | --- | --- |
|  | Free will | *Ref.* |
|  | Arranged with consent | 0.135** |
|  | Arranged without consent | 0.491*** |
| *Number of children still alive* | | 0.0661*** |
| *Number of marriages* | |  |
|  | *One* | *Ref.* |
|  | *More than one* | 0.344*** |
| *Partner's mother tongue* | |  |
|  | Turkish | *Ref.* |
|  | Kurdish | -0.041 |
|  | Arabic | -0.562*** |
|  | Other | -0.182 |
| *Nuclear family* | | -0.021 |
| *Alcohol consumption of the partner* | |  |
|  | Never | *Ref.* |
|  | Every day/almost every day | 0.682*** |
|  | Once or twice a week | 0.541*** |
|  | A few times a month | 0.398*** |
|  | Less than once a month | 0.189** |
| *Geographic area* | |  |
|  | Urban | *Ref.* |
|  | Rural | -0.0895* |
| *Region fixed effects (Nuts-1)* | | *Yes* |
| Constant |  | -1.278*** |
| N |  | 6129 |
| Pseudo R-sq | | 0.075 |

* p<0.10, ** p<0.05, *** p<0.01.

Data: Domestic Violence Survey Turkey, 2014.

Sample: ever-married women aged 15 to 59.

Simple probit model distinguishing between 3 different categories for woman’s working status (working, not allowed to work, inactivity for other reasons). Model estimated with robust standard errors, includes region fixed effects (Nuts-1).

*Appendix C: Estimated probability of female formal employment, respectively women ‘contributing at least the same than others to household income’ (bi-probit)*

*Core dependent variable: cluster average*

|  |  | Model A: Probability of being in formal employment (bi-probit) | Model B: Probability of contributing at least the same than others to household income (bi-probit) |
| --- | --- | --- | --- |
| *Cluster average* | | 0.732*** | 4.131*** |
| *Woman's education* | |  |  |
|  | Low education (pre-primary, primary) | *Ref.* | *Ref.* |
|  | Middle education (secondary) | 0.283*** | 0.150** |
|  | High education (tertiary) | 1.244*** | 0.989*** |
| *Partner's education* | |  |  |
|  | Low education (pre-primary, primary) | *Ref.* | *Ref.* |
|  | Middle education (secondary) | -0.143*** | -0.184*** |
|  | High education (tertiary) | -0.0770 | -0.174* |
| *Partner/family does not allow to work* | | / | -6.780*** |
| *Partner's labour market status* | |  |  |
|  | Working | *Ref.* | / |
|  | Not working | -0.0982 | / |
| *Woman's age* | |  |  |
|  | 15-19 | *Ref.* | *Ref.* |
|  | 20-29 | 0.263 | 0.423 |
|  | 30-39 | 0.736*** | 0.744* |
|  | 40-49 | 0.706*** | 0.835* |
|  | 50-59 | 0.490* | 0.780* |
| *Age difference with partner* | | 0.00607 | 0.00483 |
| *Age at marriage* | |  |  |
|  | 10-17 | *Ref.* | *Ref.* |
|  | 18-21 | -0.0527 | -0.097 |
|  | 22-29 | 0.0573 | -0.00864 |
|  | 30+ | -0.0179 | -0.141 |

*…*

*Appendix C continued*

| *Type of marriage* | |  |  |
| --- | --- | --- | --- |
|  | Free will | *Ref.* | *Ref.* |
|  | Arranged with consent | -0.170*** | -0.0314 |
|  | Arranged without consent | 0.0519 | 0.00947 |
| *Number of children still alive* | | -0.0861*** | -0.110*** |
| *Number of marriages* | |  |  |
|  | *One* | *Ref.* | *Ref.* |
|  | *More than one* | 0.0104 | -0.306** |
| *Partner's mother tongue* | |  |  |
|  | Turkish | *Ref.* | *Ref.* |
|  | Kurdish | -0.0764 | -0.000373 |
|  | Arabic | -0.120 | -0.241 |
|  | Other | -0.0278 | 0.223 |
| *Nuclear family* | | -0.0636 | -0.0664 |
| *Alcohol consumption of the partner* | |  |  |
|  | Never | *Ref.* | *Ref.* |
|  | Every day/almost every day | 0.411*** | 0.272** |
|  | Once or twice a week | 0.315*** | 0.0935 |
|  | A few times a month | 0.385*** | 0.195* |
|  | Less than once a month | 0.353*** | 0.260*** |
| *Current couple status* | |  |  |
|  | Married | / | *Ref.* |
|  | Widow, separated, divorced (recently) | / | 1.272*** |
| *Geographic area* | |  |  |
|  | Urban | *Ref.* | *Ref.* |
|  | Rural | 0.0145 | -0.009 |
| *Region fixed effects (Nuts-1)* | | *Yes* | *Yes* |
| Constant | | -1.646*** | -2.153*** |
| N |  | 6129 | 6129 |
| Artrho | | 0.817*** | 0.253* |
| Rho |  | 0.673 | 0.248 |
| Wald test | | 10.797*** | 3.78606* |

* p<0.10, ** p<0.05, *** p<0.01.

Data: Domestic Violence Survey Turkey, 2014.

Sample: ever-married women aged 15 to 59.

Both models are estimated with robust standard errors and include region fixed effects (Nuts-1).

*Appendix D: Estimated probability of having experienced sexual and/or physical violence by their husband/partner during the last 12 months (simple probit)*

*Core dependent variables: cluster average & woman’s labor market status, respectively woman’s contribution to household income*

|  |  | Model C | Model D |
| --- | --- | --- | --- |
| *Cluster average* | | -0.204 | -0.132 |
| *Woman's labor market status* | |  |  |
|  | Formal employment | -0.0482 | N/A |
|  | Informal/irregular employment | *Ref.* | N/A |
|  | Inactivity (other) |  | N/A |
|  | Partner does not allow to work |  | 0.325*** |
| *Woman's contribution to family income* | |  |  |
|  | About the same than others | N/A | -0.0733 |
|  | Only she has income in the household | N/A |  |
|  | More than others | N/A |  |
|  | Less than others | N/A | *Ref.* |
|  | Nothing | N/A |  |
| *Partner's labour market status* | |  |  |
|  | Working | *Ref.* | N/A |
|  | Not working | -0.0447 | N/A |
| *Woman's education* | |  |  |
|  | Low education (pre-primary, primary) | *Ref.* | *Ref.* |
|  | Middle education (secondary) | 0.107* | 0.100* |
|  | High education (tertiary) | -0.00926 | -0.00209 |
| *Partner's education* | |  |  |
|  | Low education (pre-primary, primary) | *Ref.* | *Ref.* |
|  | Middle education (secondary) | -0.147*** | -0.148*** |
|  | High education (tertiary) | -0.407*** | -0.395*** |
| *Woman's age* | |  |  |
|  | 15-19 | *Ref.* | *Ref.* |
|  | 20-29 | -0.136 | -0.129 |
|  | 30-39 | -0.369* | -0.368* |
|  | 40-49 | -0.598*** | -0.598*** |
|  | 50-59 | -0.910*** | -0.919*** |

*…*

*Appendix D continued*

| *Age difference with partner* | | -0.000505 | -0.00168 |
| --- | --- | --- | --- |
| *Age at marriage* | |  |  |
|  | 10-17 | *Ref.* | *Ref.* |
|  | 18-21 | -0.085 | -0.0774 |
|  | 22-29 | -0.074 | -0.0732 |
|  | 30+ | -0.111 | -0.115 |
| *Type of marriage* | |  |  |
|  | Free will | *Ref.* | *Ref.* |
|  | Arranged with consent | 0.130** | 0.132** |
|  | Arranged without consent | 0.498*** | 0.492*** |
| *Number of children still alive* | | 0.0596*** | 0.0660*** |
| *Number of marriages* | |  |  |
|  | *One* | *Ref.* | *Ref.* |
|  | *More than one* | 0.349*** | 0.349*** |
| *Current couple status* | |  |  |
|  | Married | N/A | *Ref.* |
|  | Widow, separated, divorced (recently) | N/A | 0.198** |
| *Partner's mother tongue* | |  |  |
|  | Turkish | *Ref.* | *Ref.* |
|  | Kurdish | -0.0385 | -0.0476 |
|  | Arabic | -0.557*** | -0.577*** |
|  | Other | -0.194 | -0.178 |
| *Nuclear family* | | -0.0183 | 0.00157 |
| *Alcohol consumption of the partner* | |  |  |
|  | Never | *Ref.* | *Ref.* |
|  | Every day/almost every day | 0.705*** | 0.650*** |
|  | Once or twice a week | 0.560*** | 0.523*** |
|  | A few times a month | 0.418*** | 0.408*** |
|  | Less than once a month | 0.203** | 0.202** |
| *Geographic area* | |  |  |
|  | Urban | *Ref.* | *Ref.* |
|  | Rural | -0.101** | -0.0766 |
| *Region fixed effects (Nuts-1)* | | *Yes* | *Yes* |
| Constant | | -1.280*** | -1.280*** |
| N |  | 6129 | 6129 |
| Pseudo R-sq | | 0.07 | 0.075 |

* p<0.10, ** p<0.05, *** p<0.01.

Data: Domestic Violence Survey Turkey, 2014.

Sample: ever-married women aged 15 to 59.

Both models are estimated with robust standard errors and include region fixed effects (Nuts-1).

*Appendix E: Estimated probability of having experienced sexual and/or physical violence by their husband/partner during the last 12 months*

*Core dependent variables: woman’s labor market status*

|  |  | Model E | Model F | Model G (bi-probit) |
| --- | --- | --- | --- | --- |
| *Woman's labor market status* | |  |  |  |
|  | Formal employment | *Ref.* | 0.0017 | -1.037*** |
|  | Informal/irregular employment | 0.1040 | *Ref.* | *Ref.* |
|  | Inactivity (incl. ‘not allowed to work’) | -0.0285 |  |  |
| *Partner's labour market status* | |  |  |  |
|  | Working | *Ref.* | *Ref.* | *Ref.* |
|  | Not working | -0.0238 | -0.0336 | -0.0484 |
| *Woman's education* | |  |  |  |
|  | Low education (pre-primary, primary) | *Ref.* | *Ref.* | *Ref.* |
|  | Middle education (secondary) | 0.130** | 0.127** | 0.204*** |
|  | High education (tertiary) | 0.0415 | 0.0409 | 0.523*** |
| *Partner's education* | |  |  |  |
|  | Low education (pre-primary, primary) | *Ref.* | *Ref.* | *Ref.* |
|  | Middle education (secondary) | -0.128** | -0.129** | -0.147*** |
|  | High education (tertiary) | -0.424*** | -0.428*** | -0.395*** |
| *Woman's age* | |  |  |  |
|  | 15-19 | *Ref.* | *Ref.* | *Ref.* |
|  | 20-29 | -0.1040 | -0.0972 | -0.0200 |
|  | 30-39 | -0.335* | -0.320* | -0.103 |
|  | 40-49 | -0.536*** | -0.522*** | -0.287 |
|  | 50-59 | -0.844*** | -0.831*** | -0.628*** |
| *Age difference with partner* | | 0.0004 | -0.0001 | 0.00125 |
| *Age at marriage* | |  |  |  |
|  | 10-17 | *Ref.* | *Ref.* | *Ref.* |
|  | 18-21 | -0.0854 | -0.0904 | -0.105* |
|  | 22-29 | -0.0793 | -0.0867 | -0.0728 |
|  | 30+ | -0.1080 | -0.1220 | -0.117 |

*…*

*Appendix E continued*

| *Type of marriage* | |  |  |  |
| --- | --- | --- | --- | --- |
|  | Free will | *Ref.* | *Ref.* | *Ref.* |
|  | Arranged with consent | 0.119** | 0.121** | 0.0526 |
|  | Arranged without consent | 0.483*** | 0.493*** | 0.443*** |
| *Number of children still alive* | | 0.0559*** | 0.0547*** | 0.0298 |
| *Number of marriages* | |  |  |  |
|  | *One* | *Ref.* | *Ref.* | *Ref.* |
|  | *More than one* | 0.381*** | 0.384*** | 0.364*** |
| *Partner's mother tongue* | |  |  |  |
|  | Turkish | *Ref.* | *Ref.* | *Ref.* |
|  | Kurdish | -0.0541 | -0.0574 | -0.0630 |
|  | Arabic | -0.573*** | -0.580*** | -0.548*** |
|  | Other | -0.1890 | -0.186 | -0.182 |
| *Nuclear family* | | -0.0382 | -0.0358 | -0.0543 |
| *Geographic area* | |  |  |  |
|  | Urban | *Ref.* | *Ref.* | *Ref.* |
|  | Rural | -0.125** | -0.102** | -0.101** |
| *Region fixed effects (Nuts-1)* | | *Yes* | *Yes* | *Yes* |
| Constant |  | -1.284*** | -1.304*** | -1.135*** |
| N |  | 6129 | 6129 | 6129 |
| Pseudo R-sq | | 0.054 | 0.052 | N/A |

* p<0.10, ** p<0.05, *** p<0.01.

Data: Domestic Violence Survey Turkey, 2014.

Sample: ever-married women aged 15 to 59.

All three models are estimated with robust standard errors and include region fixed effects (Nuts-1).

Model 1: simple probit model distinguishing between 3 different categories for woman’s working status (formal employment, informal/irregular employment, inactivity including not allowed to work).

Model 2: simple probit model distinguishing between 2 different categories for woman’s working status (formal employment, any other).

Model 3: bi-probit model distinguishing between 2 different categories for woman’s working status (formal employment, any other.

Bi-probit: seemingly unrelated bivariate probit implementing instrumental variables.

The endogenous regressor ‘woman’s formal employment’ is instrumented with the cluster average (= proportion of women in formal employment in each cluster (cluster = census enumeration area). The cluster excludes the woman being considered in each observation to avoid in-built correlation. Two binary dependent variables are estimated jointly: The probability of being in formal employment as a function of the cluster average, and the probability of experiencing domestic violence as a function of the probability of being in formal employment, while both estimations share the same list of control variables. The error terms of the two equations are allowed to be freely correlated in order to account for the possibility that some unobserved factors influence the two outcomes.

*Appendix F: Estimated probability of having experienced sexual and/or physical violence by their husband/partner during the last 12 months*

*Core dependent variables: woman’s contribution to household income & couple’s employment status*

|  |  | Model H (probit) | Model I  (probit) | Model J (bi-probit) |
| --- | --- | --- | --- | --- |
| *Woman's contribution to family income* | |  |  |  |
|  | About the same than others | *Ref.* | -0.149* | -0.360* |
|  | Only she has income in the household | 0.417** |  |  |
|  | More than others | 0.16 |  |  |
|  | Less than others | 0.242* | *Ref.* | *Ref.* |
|  | Nothing | 0.351*** |  |  |
| *Partner/family does not allow to work* | | 0.350*** | 0.356*** | 0.350*** |
| *Couple's employment status* | |  |  |  |
|  | Neither work | *Ref.* | *Ref.* | *Ref.* |
|  | Only he works | 0.0654 | 0.0729 | 0.0659 |
|  | Both work | 0.218** | 0.174** | 0.208** |
|  | Only she works | 0.275** | 0.268** | 0.317** |
| *Woman's education* | |  |  |  |
|  | Low education (pre-primary, primary) | *Ref.* | *Ref.* | *Ref.* |
|  | Middle education (secondary) | 0.102* | 0.0979 | 0.104* |
|  | High education (tertiary) | -0.00175 | -0.0197 | 0.0277 |
| *Partner's education* | |  |  |  |
|  | Low education (pre-primary, primary) | *Ref.* | *Ref.* | *Ref.* |
|  | Middle education (secondary) | -0.150*** | -0.149*** | -0.150*** |
|  | High education (tertiary) | -0.389*** | -0.398*** | -0.393*** |
| *Woman's age* | |  |  |  |
|  | 15-19 | *Ref.* | *Ref.* | *Ref.* |
|  | 20-29 | -0.132 | -0.138 | -0.13 |
|  | 30-39 | -0.379* | -0.388* | -0.373* |
|  | 40-49 | -0.605*** | -0.614*** | -0.595*** |
|  | 50-59 | -0.906*** | -0.919*** | -0.897*** |
| *Age difference with partner* | | -0.000905 | -0.000962 | -0.000702 |
| *Age at marriage* | |  |  |  |
|  | 10-17 | *Ref.* | *Ref.* | *Ref.* |
|  | 18-21 | -0.0773 | -0.0725 | -0.0731 |
|  | 22-29 | -0.0682 | -0.0677 | -0.0668 |
|  | 30+ | -0.0998 | -0.101 | -0.0996 |

*…*

*Appendix D continued*

| *Type of marriage* | |  |  |  |
| --- | --- | --- | --- | --- |
|  | Free will | *Ref.* | *Ref.* | *Ref.* |
|  | Arranged with consent | 0.131** | 0.136** | 0.133** |
|  | Arranged without consent | 0.487*** | 0.485*** | 0.479*** |
| *Number of children still alive* | | 0.0659*** | 0.0677*** | 0.0652*** |
| *Number of marriages* | |  |  |  |
|  | *One* | *Ref.* | *Ref.* | *Ref.* |
|  | *More than one* | 0.339*** | 0.341*** | 0.331*** |
| *Partner's mother tongue* | |  |  |  |
|  | Turkish | *Ref.* | *Ref.* | *Ref.* |
|  | Kurdish | -0.0402 | -0.0385 | -0.0355 |
|  | Arabic | -0.571*** | -0.569*** | -0.565*** |
|  | Other | -0.174 | -0.182 | -0.181 |
| *Nuclear family* | | -0.00042 | -0.00177 | -0.00447 |
| *Alcohol consumption of the partner* | |  |  |  |
|  | Never | *Ref.* | *Ref.* | *Ref.* |
|  | Every day/almost every day | 0.638*** | 0.639*** | 0.645*** |
|  | Once or twice a week | 0.528*** | 0.518*** | 0.519*** |
|  | A few times a month | 0.399*** | 0.394*** | 0.399*** |
|  | Less than once a month | 0.200** | 0.190** | 0.198** |
| *Current couple status* | |  |  |  |
|  | Married | *Ref.* | *Ref.* | *Ref.* |
|  | Widow, separated, divorced (recently) | 0.138 | 0.208** | 0.283** |
| *Geographic area* | |  |  |  |
|  | Urban | *Ref.* | *Ref.* | *Ref.* |
|  | Rural | -0.102* | -0.0887* | -0.100* |
| *Region fixed effects (Nuts-1)* | | *Yes* | *Yes* | *Yes* |
| Constant | | -1.777*** | -1.448*** | -1.433*** |
| N |  | 6129 | 6129 | 6129 |
| Pseudo R-sq | | 0.078 | 0.076 | N/A |

* p<0.10, ** p<0.05, *** p<0.01.

Data: Domestic Violence Survey Turkey, 2014.

Sample: ever-married women aged 15 to 59.

All three models are estimated with robust standard errors and include region fixed effects (Nuts-1).

Model E: simple probit model distinguishing between 5 different categories for woman’s contribution to household income: about the same, all, more, less, nothing.

Model F: simple probit model distinguishing between 2 different categories for woman’s contribution to household income: at least the same (combining about the same, all and more) against less or nothing (combined).

Model G: bi-probit model distinguishing between 2 different categories for woman’s contribution to household income: at least the same (combining about the same, all and more) against less or nothing (combined).

Bi-probit: seemingly unrelated bivariate probit implementing instrumental variables.

The endogenous regressor ‘woman contributing at least the same’ is instrumented with the cluster average (= proportion of women contributing at least the same in each cluster (cluster = census enumeration area). The cluster excludes the woman being considered in each observation to avoid in-built correlation. Two binary dependent variables are estimated jointly: The probability of ‘contributing at least the same’ as a function of the cluster average, and the probability of experiencing domestic violence as a function of the probability of ‘contributing at least the same’, while both estimations share the same list of control variables. The error terms of the two equations are allowed to be freely correlated in order to account for the possibility that some unobserved factors influence the two outcomes.

*Appendix G: Estimated probability of having experienced violence by the last husband/partner during the last 12 month – by types of violence, probit regression with robust standard errors*

|  |  | only physical | only sexual | pysical & sexual | emotional | economic |  | only physical | only sexual | pysical & sexual | emotional | economic |
| --- | --- | --- | --- | --- | --- | --- | --- | --- | --- | --- | --- | --- |
| *Woman's labor market status* | |  |  |  |  |  |  |  |  |  |  |  |
|  | Formal employment | *Ref.* | *Ref.* | *Ref.* | *Ref.* | *Ref.* |  | / | / | / | / | / |
|  | Informal/irregular employment | 0.116 | 0.269* | -0.0402 | 0.107 | 0.340*** |  | / | / | / | / | / |
|  | Inactivity (other) | 0.0502 | 0.0215 | -0.168 | -0.00156 | 0.0683 |  | / | / | / | / | / |
|  | Partner does not allow to work | 0.267** | 0.352** | 0.152 | 0.359*** | 0.950*** |  | / | / | / | / | / |
| *Partner's labour market status* | |  |  |  |  |  |  |  |  |  |  |  |
|  | Working | *Ref.* | *Ref.* | *Ref.* | *Ref.* | *Ref.* |  | / | / | / | / | / |
|  | Not working | -0.155* | 0.0568 | 0.116 | 0.0313 | 0.0258 |  | / | / | / | / | / |
| *Partner/family does not allow to work* | | / | / | / | / | / |  | 0.212** | 0.267** | 0.296** | 0.328*** | 0.836*** |
| *Woman's contribution to family income* | |  |  |  |  |  |  |  |  |  |  |  |
|  | About the same than others | / | / | / | / | / |  | *Ref.* | *Ref.* | *Ref.* | *Ref.* | *Ref.* |
|  | Only she has income in the household | / | / | / | / | / |  | 0.137 | 0.791** | 0.463* | 0.201 | 0.391** |
|  | More than others | / | / | / | / | / |  | 0.0606 | 0.567 | 0.0780 | 0.0433 | 0.128 |
|  | Less than others | / | / | / | / | / |  | 0.130 | 0.455 | 0.253 | 0.209** | 0.368*** |
|  | Nothing | / | / | / | / | / |  | 0.0987 | 0.495* | 0.265 | 0.185** | 0.284** |
| *Woman's education* | |  |  |  |  |  |  |  |  |  |  |  |
|  | Low education (pre-primary, primary) | *Ref.* | *Ref.* | *Ref.* | *Ref.* | *Ref.* |  | *Ref.* | *Ref.* | *Ref.* | *Ref.* | *Ref.* |
|  | Middle education (secondary) | 0.112 | 0.0430 | 0.0331 | 0.154*** | 0.0314 |  | 0.111 | 0.0484 | 0.0208 | 0.159*** | 0.0252 |
|  | High education (tertiary) | -0.0997 | 0.131 | 0.111 | -0.0224 | -0.241** |  | -0.105 | 0.139 | 0.137 | 0.0129 | -0.245** |
| *Partner's education* | |  |  |  |  |  |  |  |  |  |  |  |
|  | Low education (pre-primary, primary) | *Ref.* | *Ref.* | *Ref.* | *Ref.* | *Ref.* |  | *Ref.* | *Ref.* | *Ref.* | *Ref.* | *Ref.* |
|  | Middle education (secondary) | -0.0686 | -0.103 | -0.232*** | -0.0774* | -0.00745 |  | -0.0662 | -0.111 | -0.227*** | -0.0858** | -0.0157 |
|  | High education (tertiary) | -0.207** | -0.629*** | -0.430*** | -0.232*** | -0.241*** |  | -0.201* | -0.642*** | -0.414*** | -0.239*** | -0.249*** |

*Appendix G continued*

| *Woman's age* | |  |  |  |  |  |  |  |  |  |  |  |
| --- | --- | --- | --- | --- | --- | --- | --- | --- | --- | --- | --- | --- |
|  | 15-19 | *Ref.* | *Ref.* | *Ref.* | *Ref.* | *Ref.* |  | *Ref.* | *Ref.* | *Ref.* | *Ref.* | *Ref.* |
|  | 20-29 | -0.0213 | -0.0988 | -0.295 | -0.00687 | -0.318* |  | -0.0178 | -0.0667 | -0.328 | 0.0137 | -0.296* |
|  | 30-39 | -0.268 | -0.112 | -0.481 | -0.0438 | -0.302* |  | -0.269 | -0.0635 | -0.528* | -0.00618 | -0.266 |
|  | 40-49 | -0.505** | -0.134 | -0.777** | -0.193 | -0.655*** |  | -0.514** | -0.0811 | -0.798*** | -0.156 | -0.623*** |
|  | 50-59 | -0.594** | -0.433 | -1.290*** | -0.403** | -0.982*** |  | -0.632*** | -0.366 | -1.318*** | -0.360* | -0.941*** |
| *Age difference with partner* | | -0.00125 | 0.00338 | -0.00257 | 0.00126 | 0.00159 |  | -0.00216 | 0.00251 | -0.00365 | 0.00159 | 0.000913 |
| *Age at marriage* | |  |  |  |  |  |  |  |  |  |  |  |
|  | 10-17 | *Ref.* | *Ref.* | *Ref.* | *Ref.* | *Ref.* |  | *Ref.* | *Ref.* | *Ref.* | *Ref.* | *Ref.* |
|  | 18-21 | -0.0753 | -0.0414 | -0.0274 | 0.0123 | 0.016 |  | -0.0752 | -0.0621 | -0.0343 | 0.00321 | 0.00536 |
|  | 22-29 | -0.0625 | -0.129 | 0.0355 | -0.0138 | -0.0871 |  | -0.0643 | -0.140 | 0.0300 | -0.0223 | -0.104 |
|  | 30+ | -0.0615 | -0.0122 | -0.187 | -0.12 | 0.133 |  | -0.0784 | -0.0534 | -0.197 | -0.126 | 0.108 |
| *Type of marriage* | |  |  |  |  |  |  |  |  |  |  |  |
|  | Free will | *Ref.* | *Ref.* | *Ref.* | *Ref.* | *Ref.* |  | *Ref.* | *Ref.* | *Ref.* | *Ref.* | *Ref.* |
|  | Arranged with consent | 0.0345 | 0.0228 | 0.388*** | 0.214*** | 0.158*** |  | 0.0351 | 0.0200 | 0.384*** | 0.213*** | 0.163*** |
|  | Arranged without consent | 0.185* | 0.326*** | 0.784*** | 0.605*** | 0.425*** |  | 0.182* | 0.362*** | 0.783*** | 0.622*** | 0.456*** |
| *Number of children still alive* | | 0.0328 | 0.0685*** | 0.0605** | 0.0205 | 0.0308* |  | 0.0330 | 0.0594** | 0.0664** | 0.0148 | 0.0251 |
| *Number of marriages* | |  |  |  |  |  |  |  |  |  |  |  |
|  | *One* | *Ref.* | *Ref.* | *Ref.* | *Ref.* | *Ref.* |  | *Ref.* | *Ref.* | *Ref.* | *Ref.* | *Ref.* |
|  | *More than one* | 0.340*** | -0.0796 | 0.440** | 0.367*** | 0.158 |  | 0.332*** | -0.0615 | 0.446** | 0.366*** | 0.151 |
| *Partner's mother tongue* | |  |  |  |  |  |  |  |  |  |  |  |
|  | Turkish | *Ref.* | *Ref.* | *Ref.* | *Ref.* | *Ref.* |  | *Ref.* | *Ref.* | *Ref.* | *Ref.* | *Ref.* |
|  | Kurdish | -0.164* | 0.0730 | 0.0652 | -0.141** | 0.107 |  | -0.178* | 0.0677 | 0.0665 | -0.140** | 0.105 |
|  | Arabic | -0.243 | N/A | -0.490 | -0.399*** | -0.246 |  | -0.248 | N/A | -0.541 | -0.399*** | -0.253 |
|  | Other | -0.454 | -0.115 | 0.296 | -0.344* | 0.127 |  | -0.459 | -0.0753 | 0.320 | -0.341* | 0.142 |
| *Nuclear family* | | 0.0262 | -0.0795 | -0.0509 | 0.104** | 0.0223 |  | 0.0374 | -0.0870 | 0.0279 | 0.0822** | 0.0109 |

*…*

*Appendix G continued*

| *Alcohol consumption of the partner* | |  |  |  |  |  |  |  |  |  |  |  |
| --- | --- | --- | --- | --- | --- | --- | --- | --- | --- | --- | --- | --- |
|  | Never | *Ref.* | *Ref.* | *Ref.* | *Ref.* | *Ref.* |  | *Ref.* | *Ref.* | *Ref.* | *Ref.* | *Ref.* |
|  | Every day/almost every day | 0.607*** | 0.238 | 0.610*** | 0.546*** | 0.815*** |  | 0.594*** | 0.298 | 0.480*** | 0.617*** | 0.864*** |
|  | Once or twice a week | 0.318*** | 0.408*** | 0.593*** | 0.502*** | 0.348*** |  | 0.315*** | 0.420*** | 0.546*** | 0.526*** | 0.354*** |
|  | A few times a month | 0.276** | 0.200 | 0.480*** | 0.456*** | 0.112 |  | 0.278** | 0.224 | 0.469*** | 0.478*** | 0.12 |
|  | Less than once a month | 0.138 | 0.0636 | 0.288** | 0.272*** | 0.075 |  | 0.139 | 0.0894 | 0.297** | 0.288*** | 0.0831 |
| *Current couple status* | |  |  |  |  |  |  |  |  |  |  |  |
|  | Married | / | / | / | / | / |  | *Ref.* | *Ref.* | *Ref.* | *Ref.* | *Ref.* |
|  | Widow, separated, divorced (recently) | / | / | / | / | / |  | 0.0117 | -0.253 | 0.446*** | -0.192** | -0.163 |
| *Geographic area* | |  |  |  |  |  |  |  |  |  |  |  |
|  | Urban | *Ref.* | *Ref.* | *Ref.* | *Ref.* | *Ref.* |  | *Ref.* | *Ref.* | *Ref.* | *Ref.* | *Ref.* |
|  | Rural | -0.0811 | -0.0601 | -0.0790 | -0.112** | -0.173*** |  | -0.0707 | -0.0252 | -0.0497 | -0.104** | -0.136*** |
| *Region fixed effects (Nuts-1)* | | *Yes* | *Yes* | *Yes* | *Yes* | *Yes* |  | *Yes* | *Yes* | *Yes* | *Yes* | *Yes* |
| Constant | | -1.780*** | -1.736*** | -2.257*** | -1.118*** | -1.072*** |  | -1.830*** | -2.173*** | -2.677*** | -1.276*** | -1.246*** |
| N |  | 6129 | 6014 | 6129 | 6129 | 6129 |  | 6129 | 6014 | 6129 | 6129 | 6129 |
| Pseudo R-sq | | 0.057 | 0.078 | 0.112 | 0.056 | 0.1 |  | 0.056 | 0.078 | 0.122 | 0.057 | 0.098 |

* p<0.10, ** p<0.05, *** p<0.01.

Data: Domestic Violence Survey Turkey, 2014.

Sample: ever-married women aged 15 to 59.
